# Learning Biomarker Models for Progression Estimation of Alzheimer’s Disease

**DOI:** 10.1371/journal.pone.0153040

**Published:** 2016-04-20

**Authors:** Alexander Schmidt-Richberg, Christian Ledig, Ricardo Guerrero, Helena Molina-Abril, Alejandro Frangi, Daniel Rueckert, on behalf of the Alzheimer’s Disease Neuroimaging Initiative

**Affiliations:** 1 Biomedical Image Analysis Group, Imperial College London, London, United Kingdom; 2 Center for Computational Imaging and Simulation Technologies in Biomedicine (CISTIB), University of Sheffield, Sheffield, United Kingdom; Indiana University, UNITED STATES

## Abstract

Being able to estimate a patient’s progress in the course of Alzheimer’s disease and predicting future progression based on a number of observed biomarker values is of great interest for patients, clinicians and researchers alike. In this work, an approach for disease progress estimation is presented. Based on a set of subjects that convert to a more severe disease stage during the study, models that describe typical trajectories of biomarker values in the course of disease are learned using quantile regression. A novel probabilistic method is then derived to estimate the current disease progress as well as the rate of progression of an individual by fitting acquired biomarkers to the models. A particular strength of the method is its ability to naturally handle missing data. This means, it is applicable even if individual biomarker measurements are missing for a subject without requiring a retraining of the model. The functionality of the presented method is demonstrated using synthetic and—employing cognitive scores and image-based biomarkers—real data from the ADNI study. Further, three possible applications for progress estimation are demonstrated to underline the versatility of the approach: classification, construction of a spatio-temporal disease progression atlas and prediction of future disease progression.

## Introduction

Alzheimer’s disease (AD), the most common cause of dementia, is a dynamic neurodegenerative condition of which roughly 36 million patients suffer today. With the ageing population, the worldwide prevalence is expected to rise to over 100 million by 2050 [1]. Patients diagnosed with Mild Cognitive Impairments (MCI)—an early form of dementia—show first symptoms of general memory loss. In the course of the disease, these symptoms are followed by behavioural change and further cognitive and functional decline, such that patients become less and less able to perform even basic tasks. As studies suggest, the annual conversion rate from MCI to AD lies between 10 and 15% [2]. However, there is considerable variability in the disease progression between patients diagnosed with dementia [3]. Clinicians, researchers, and patients alike therefore often face two questions: How severe is the form of dementia? And how fast will the progression of the disease be?

Estimating the current stage of the disease and predicting the rate of progression has been the aim of many publications. Most approaches consider results of cognitive tests like the Mini—mental State Examination (MMSE), the Alzheimer’s Disease Assessment Scale (ADAS), and the Clinical Dementia Rating (CDR) as biomarkers that characterise disease progression. For example, Doody et al. use the measured MMSE score together with the estimated duration of symptoms to compute a pre-progression rate, that means the rate of decline prior to the first physician visit [4]. Test subjects are then grouped in slow, intermediate and rapid progression. Based on this, the future course of the disease is predicted [3].

In many approaches—see for example [5] for a detailed overview—Markov transition models and Cox proportional hazard model are employed to estimate disease progression. For example, Neumann et al. [6] and Spackman et al. [7] estimate the probability of a transition between discrete CDR states based on the Behaviour Rating Scale for Dementia (BRSD) while considering explanatory variables like age, sex and the mental status of the patients. Similarly, in the model proposed by Caro et al. [8], the probability of a patient needing full-time care or dying within a given period of time is estimated using a Markov model with time dependent hazard functions. However, as Green et al. conclude in their review, the main limitations of these methods are “the use of a limited number of health states to capture events related to disease progression over time” and the fact that “a single symptom, such as cognition, is not able to characterise AD progression” [5]. Stallard et al. further argue from a methodological point of view that varying progression rates violate assumptions of Markov transition models and that Cox regression models implicitly assume the predictors to be fixed for individual patients (which, e.g., age and cognitive function are not) [9]. They therefore propose a longitudinal Grade of Membership (L-GoM) model, in which MMSE, BRSD, education, alcohol use, and other attributes are used to estimate the disease progression in three separate dimensions (cognitive, clinical, and functional). This approach is further evaluated by Razlinghi et al. [10] to predict the time to events such as full-time care, institutionalisation, or death.

Another important development in the field of progression estimation aims at deriving meaningful biomarkers from image data to capture pathological changes of the brain structure. This approach is based on the observation that neurodegeneration precedes clinical symptoms [11] and therefore holds valuable information about early disease progression. For example, Moradi et al. give a comprehensive overview on machine learning methods in which imaging biomarkers are used to predict conversion from one disease stage to another [12]. Hua et al. show that neuroimaging measures derived from tensor-based morphometry (TBM) can considerably reduce the sample size required for progression estimation [13]. Sabuncu et al. use an extended Cox regression model to associate longitudinal biomarkers derived from MRI data with the occurrence of clinical events [14]. In [15], a brain age gap estimation (BrainAGE) score is computed based on typical atrophy patterns in MR images and employed to predict conversion from MCI to AD. Another interesting approach is presented by Fonteijin et al. [16] and later extended by Young et al. [17]. Here, an event-based model is used to determine the order in which cerebrospinal fluid, image-based and cognitive biomarkers become abnormal. This information can then be used to assign a subject to one of several discrete disease stages.

However, modelling disease progression by a number of discrete stages is a simplification of a continuous process and entails some limitations. Jack et al., for example, argue that “evolving diagnostic criteria must incorporate AD biomarkers but cannot proceed effectively without accurate time-dependent models of those biomarkers” [18]. Availability of further knowledge about the temporal offset between discrete states also simplifies the prediction of cognitive decline within a given period of time. To this end, some approaches have been developed that regard the course of disease as a continuous progress. For example, Jack et al. analyse the shape of typical biomarker trajectories depending on the MMSE score of the subject [19]. Yang et al. assume an exponential-shaped trajectory of the ADAS score [20]. Based on the fact that study entry time does not correspond to the start of the disease, they then estimate a temporal offset *γ* indicating the disease progress of a subject by fitting its ADAS scores to this curve. Similarly, Delor et al. [21] compute a *disease onset time* (DOT) by adjusting subjects according to their CDR-SB (Sum of Boxes) score. While these methods make strong assumptions on the trajectories’ shapes, Donohue et al. only assume them to be smooth and monotone [22]. Shape parameters and temporal offset are then estimated in an alternating fashion by minimising the residual sum of squares.

Rather than only focusing on the mean trajectory—as done in least squares regression—quantile regression models the median and other quantile functions of a response variable in dependency of the predictor variable [23]. The response is not required to be normal distributed, which is of particular interest for variables that show boundary effects (as, for example, many cognitive test scores). In the context of Alzheimer’s disease, Sherwood et al. recently proposed linear quantile regression to estimate the conditional percentiles of neuropsychological test scores [24]. Li et al. model the episodic memory outcome (a composite score comprised of six clinical neuropsychological tests) with age and time to AD diagnosis as covariates [25]. Their model is also linear but allows a single change-point at which the slope of the response variable changes rapidly. While this assumption is reasonable for cognitive function, it is unclear to which degree it holds for imaging-based biomarkers. Therefore, Schmidt-Richberg et al. proposed to employ Vector Generalised Additive Models (VGAM’s) for quantile regression [26]. VGAM’s are based on vector smoothers, which means that smoothness is the only assumption made on the shape of the curves. In contrast to [22], non-monotonic trajectories (as arising, for example, from manifold learning) are allowed. However, application of this technique requires a prior temporal alignment of the subjects. Similarly to [25], this is done based on the time to AD diagnoses.

This work builds upon the preliminary results presented in [26] and employs VGAM’s vor model estimation. Utilising the estimated quantile curves, a probabilistic model is derived to compute a *time warp* that indicates the disease progress and the rate of progression of a subject based on measured biomarker values. The approach is flexible with regard to the considered biomarkers, which can be based on cognitive scores or neuroimaging, for instance. Moreover, missing data is handled in a natural way. This means, the approach can be employed even if the set of observed biomarkers is not complete. This work also adds a thorough evaluation of the approach based on synthetic and real data to the results presented in [26]. Further, the applicability is demonstrated for classification tasks, 4D atlas generation, as well as the prediction of future biomarker values.

## Methods

The presented approach of disease progression analysis is based on a set of quantitative measurements of variables that have the potential to characterise disease progression. These measurements could be biological markers (for example A*β* plaque deposition), results from cognitive and functional tests, volumes of brain structures or any other kind of features derived from medical imaging techniques. For the sake of simplicity, we refer to these measurements as *biomarkers* (in an extended meaning of the term) in the remainder of this article.

Let $y_{sv}^{b}$ denote biomarker values acquired from multiple subjects *s* ∈ *S* = [1, …, *n*_*S*_] during multiple visits *v* ∈ *V*_*s*_, with *b* ∈ *B*_*sv*_ denoting the biomarker index. Each biomarker vector is associated with the time $t_{sv}\in T=R^{+}$ of acquisition, measured in days after the first (baseline) visit *t*_*s*1_ = 0, as well as the diagnosis *d*_*sv*_ that was given during each visit. In this work, *d*_*sv*_ ∈ {*CN*, *EMCI*, *LMCI*, *AD*} is assumed with CN denoting cognitively normal subjects and EMCI and LMCI early and late MCI, respectively. It is to be noted that the number of visits can vary for each subject, *V*_*s*_ ⊆ *V* = [1, …, *n*_*V*_]. Also, the biomarkers acquired at each visit might differ, such that *B*_*sv*_ ⊆ *B* = [1, …, *n*_*B*_].

The proposed method consists of two parts. In the first phase (following machine learning methods referred to as training phase), “typical” trajectories of biomarkers in the course of disease progression are determined based on the values measured for a number of training subjects. In the second phase, these models are employed to estimate how far test subjects have progressed along the disease trajectory.

### Model training

The model training phase aims at learning typical trajectories of biomarker values in the course of the disease. Instead of just concentrating on the mean trajectories, also the variations of biomarker values in the cohort are of interest. That means, the probability that a certain biomarker *b* has a value *y*^*b*^ at a specified time point in the disease is to be determined. More technically, each measured biomarker value $y_{sv}^{b}$ is understood as an observation of a dependent variable *Y*^*b*^ given an explanatory variable or covariate *p*_*sv*_. Here, *p*_*sv*_ ∈ *P* is called the *disease progress*, which indicates how far the subject *s* has progressed in the continuum of the disease *P* at the time of visit *v*. The notation of a *disease progress* is chosen over *disease stage* (cf. [17]) to emphasise that the course of the disease is seen as a continuous progress rather than a sequence of discrete stages. Thus, *p*_*sv*_ can be seen as an offset to some specified time point *p* = 0, for example disease onset. In this work, P⊆R is simplistically assumed to be one-dimensional. The probability density function (PDF) of *Y*^*b*^ given *p* is then denoted by *f*_*Y*^*b*^_ (*y*|*p*).

A *disease progression model* M(p) comprises the density functions of all biomarkers in *B* on a domain *P* and is defined as

$$\begin{array}{c} M\left(p\right)=\left\{M^{1}\left(p\right),\cdots ,M^{n_{B}}\left(p\right)\right\}\;\text{with}\;M^{b}\left(p\right):=f_{Y^{b}}\left(y|p\right) \end{array}$$ (1)

for *p* ∈ *P*. Another way of representing the model is by its *q*-quantile functions

$$\begin{array}{c} y_{q}^{b}\left(p\right):=\inf\left\{y\in R:F_{Y^{b}}\left(y\;|\;p\right)\leq q\right\},\;q\in \left(0,1\right), \end{array}$$

where *F*_*Y*^*b*^_ (*y*|*p*) is the cumulative distribution function corresponding to *f*_*Y*^*b*^_ (*y*|*p*). For example, $y_{0.5}^{b}\left(p\right)$ denotes the median trajectory. Moreover, *q*^*b*^(*y*, *p*) is defined as the function that maps a biomarker value *y* to the corresponding quantile at disease progress *p*.

The model training consists of two main steps. First, the training subjects have to be temporally aligned to establish correspondences between the time points of observation. Based on the aligned data, progression models are then estimated using quantile regression to learn the PDF *f*_*Y*^*b*^_ (*y*|*p*).

#### Temporal alignment of the training data

The temporal alignment aims at associating the time points *t*_*sv*_ of biomarker acquisition (measured in days after baseline) to the corresponding disease progresses *p*_*sv*_. In detail, the goal is to find a strict monotonically increasing (and thereby order preserving) function *τ*(*t*) that maps the subject-specific acquisition time *t*_*sv*_ ∈ *T* to the population-based disease progression time *p*_*sv*_ ∈ *P*, such that *p*_*sv*_ = *τ*(*t*_*sv*_). Following [27], *τ*(*t*) is denoted as *time warp* in this work.

The main challenge of the model training is that the real progression of training subjects is unknown. Therefore, the time point at which a more severe disease stage is first diagnosed by a clinician is considered as indicator for the disease progress. In particular, the point of conversion $t_{s}^{0}$ at which the diagnosis of subject *s* changes from MCI to AD is set to *p* = 0, that means

$$\begin{array}{c} p_{sv}=\tau \left(t_{s}^{0};t_{sv}\right):=t_{sv}-t_{s}^{0} \end{array}$$ (2)

For this reason, the training set consists of all subjects that convert from one diagnosis to another during the study (here, from MCI to AD). To identify $t_{s}^{0}$, the index *κ* is determined, such that *v*^*κ*^ and *v*^*κ*+1^ are the visits with the last MCI and the first AD diagnosis, i.e. *d*_*sv*^*κ*^_ = MCI, *d*_*sv*^*κ*+1^_ = AD. The time point of conversion is then assumed to be the average of these two visits:

$$\begin{array}{c} t_{s}^{0}:=\frac{t_{sv^{\kappa }}+t_{sv^{\kappa +1}}}{2}. \end{array}$$

**Fig 1:**
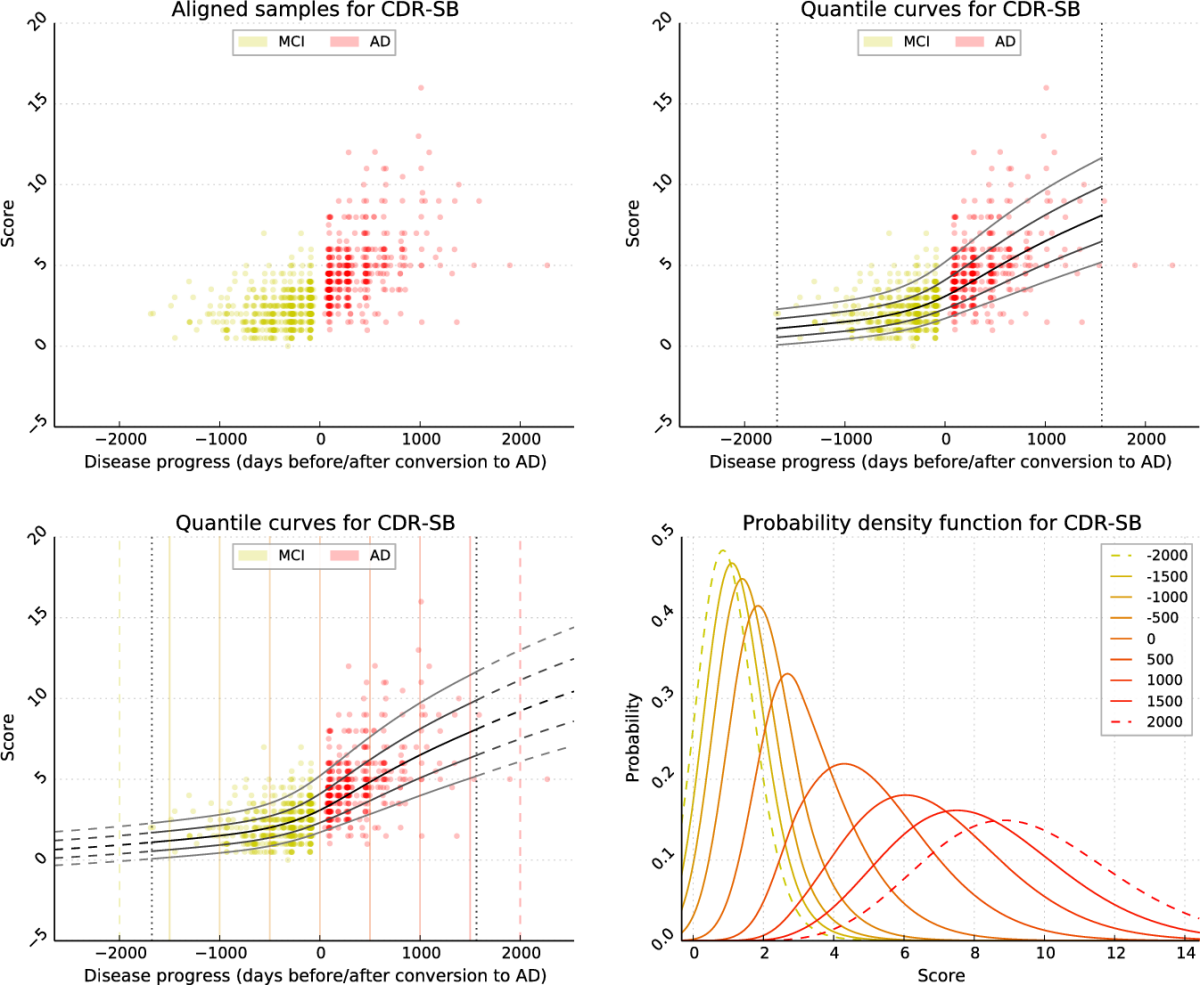
Illustration of the model training process on the example of the CDR-SB cognitive score. (A) First, sample points are temporally aligned according to the point on conversion. The colours indicate MCI (yellow) or AD (red) diagnosis. (B) The progression model is then estimated using quantile regression. The quantile functions $y_{q}^{b}\left(p\right)$ with *q* ∈ {0.1, 0.25, 0.5, 0.75, 0.9} are visualised. (C) To increase the domain *P*, the model is then extrapolated. For each solid vertical line, the corresponding PDF is given in Fig 1D. (D) Illustration of the corresponding density functions *f*_*Y*^*b*^_ (*y*|*p*), that indicate the probability of values *y* at given progresses *p*.

The set of all aligned training samples is illustrated exemplarily for the CDR-SB in Fig 1A. In the current model, training subjects are assumed to progress with the same rate in the course of disease.

#### Model training using VGAM

The aim of this section is to estimate the conditional density functions *f*_*Y*^*b*^_ for arbitrary biomarkers. In this context, Jack et al. assume a sigmoidal shape of functional, biological and imaging-based biomarkers [11]. Donohue et al., however, rather observe linear or near-linear shapes of several trajectories [22]. Other biomarkers, such as coordinates obtained from manifold learning, follow arbitrary trajectories not even necessarily monotonic [28]. In contrast to logistic or exponential regression as employed—for example—in [20], the approach followed in this work aims to make no assumptions on the shape of biomarker trajectories besides smoothness and thereby allows to treat all biomarkers in a unified way. To this end, quantile curves are estimated via *vector generalised additive models* (VGAMs) [29].

Technically, the goal of VGAMs is to estimate *f*_*Y*_ (*y*|*p*), the conditional probability density function (PDF) of a dependent variable *Y* given an explanatory variable (or covariate) *p*. It is assumed that *f*_*Y*_ can be described by some smooth function *g*_*Y*_ with

$$\begin{array}{c} f_{Y}\left(y\;|\;p\right)=g_{Y}\left(y,\eta_{1}\left(p\right),\cdots ,\eta_{M}\left(p\right)\right). \end{array}$$

Here, *η*_*m*_ are the *predictors* given by

$$\begin{array}{c} \eta_{m}\left(p\right)=\beta_{m}+f_{m}\left(p\right), \end{array}$$

where *β*_*m*_ are intercept values and *f*_*m*_ are smooth functions of the covariate estimated by cubic smoothing splines, or *vector smoothers* [30]. In contrast to parametric *vector generalised linear models* (VGLM), where *η*_*m*_(*p*) = *β*_*m*_0__ + *β*_*m*_1__ *p* are linear predictors, the relation between the *p* and *η* is only constrained to be smooth in additive models. This allows a more flexible estimation of the trajectories by capturing non-linear features of the data.

To model a normal distribution of *Y*, *g*_*Y*_ could be given by a Gaussian function with *η*_1_ being the mean and *η*_2_ the standard deviation. However, some biomarkers regarded in this work cannot take negative values and the corresponding density functions are therefore likely to be skewed. Therefore, the LMS (Lambda, Mu, Sigma) method by Cole et al. [31] is employed for quantile regression. Here, skewness is allowed by applying the Yeo-Johnson transformation *ψ*(*y*, *λ*) with

$$\begin{array}{c} \psi \left(y,\lambda \right)=\left\{ \begin{array}{cc} ({\left(y+1\right)}^{\lambda })/\lambda & \left(y\geq 0,\lambda \neq 0\right)\\ \log(y+1) & \left(y\geq 0,\lambda =0\right)\\ -\left({\left(-y+1\right)}^{2-\lambda }-1\right)/\left(2-\lambda \right) & \left(y<0,\lambda \neq 2\right)\\ -\log(-y+1) & \left(y<0,\lambda =2\right) \end{array}\right. \end{array}$$

on the response, such that the values of the transformed dependent variable approximate normality [32]. The density function *g*_*Y*_ at *p* then depends on three predictors *λ*(*p*) := *η*_1_(*p*), *μ*(*p*) := *η*_2_(*p*), and *σ*(*p*) := *η*_3_(*p*), and is given by

$$\begin{array}{ccc} g_{Y}\left(y,\lambda \left(p\right),\mu \left(p\right),\sigma \left(p\right)\right) & = & \frac{1}{\sigma (p)}\phi \left(\frac{\psi (y,\lambda (p))-\mu (p)}{\sigma (p)}\right)\\ & & \;\cdot {\left(|y|+1\right)}^{\text{sgn}(y)(\lambda (p)-1)} \end{array}$$

with *ϕ*(⋅) being a standard normal distribution. The corresponding *q*-quantile curves are then given by

$$\begin{array}{c} y_{q}\left(p\right)=\psi^{-1}\left(\lambda \left(p\right),\mu \left(p\right)+Z_{q}\sigma \left(p\right)\right). \end{array}$$ (3)

Here, the factor *Z*_*q*_ := Φ^−1^(*q*) relates the quantile *q* ∈ (0, 1) to the corresponding standard deviation *σ* using the percent point function (PPF) *ϕ*^−2^, which is the inverse of the cumulative distribution function (CDF) Φ of a normal distribution. For example, the *q* = 0.5 quantile corresponds to the median with *Z*_0.5_ = 0. A thorough and more formal introduction to VGAMs can be found in [29]. The implementation is based on the R package ‘VGAM’ (http://cran.r-project.org/web/packages/VGAM/index.html).

One particular drawback of using VGAMs is that the domain *P* of $M^{b}\left(p\right)$ is limited to the progress interval contained in the sample set. This means *P* is given by *P* = [*p*_−_, *p*_+_], with *p*_−_ := min_*s*, *v*_(*p*_*sv*_) and *p*_+_ := max_*s*, *v*_(*p*_*sv*_) being the earliest and latest observed progress, respectively. Therefore, an approach to extrapolate the model by extending the underlying predictor functions is presented in appendix A1 and visualised in Fig 1C. As a result, P=R is assumed in the following.

The estimated percentile curves obtained by VGAM and the corresponding probability density functions of the CDR-SB are exemplarily visualised in Fig 1B and 1D, respectively.

### Progress estimation

Generally, the point of conversion $t_{s}^{0}$ is unknown because it is not contained in the set of observations (or—in case of healthy and MCI subjects—has not yet been reached) and Eq (2) cannot be employed. Progress estimation is therefore understood as the task of finding the most likely time warp *τ*(*t*) that optimally fits the biomarkers values measured for a test subject into the model M.

Let ***t***_*s*_ = (*t*_*s*1_, …, *t*_*sn*_*V*__)^*T*^ be the vector containing the time points of all visits of subject *s* and *τ*(***t***_*s*_) = (*τ*(*t*_*s*1_), …, *τ*(*t*_*sn*_*V*__))^*T*^. Let further ***y***_*s*_ = (***y***_*sv*_)_*v* ∈ *V*_*s*__ be the vector with the biomarkers measured for *s* with $y_{sv}={\left(y_{sv}^{b}\right)}_{b\in B_{sv}}$ denoting the biomarkers acquired at visit *v*. Based on ***t***_*s*_, the most probable time warp ${\hat{\tau }}_{s}$ given ***y***_*s*_ is determined by maximising the logarithm of the likelihood function $L(\tau \left(t_{s}\right)\;|\;y_{s})$. This means

$$\begin{array}{c} {\hat{\tau }}_{s}:=\underset{\tau }{\text{argmax}}\log L\left(\tau \left(t_{s}\right)\;|\;y_{s}\right)=\underset{\tau }{\text{argmax}}\log f_{Y}\left(y_{s}\;|\;\tau \left(t_{s}\right)\right) \end{array}$$ (4)

with $Y=(Y^{1},\cdots ,Y^{n_{B}})$. The joint probability of all observations $y_{sv}^{b}$ given *τ* is then

$$\begin{array}{c} f_{Y}\left(y_{s}\;|\;\tau \left(t_{s}\right)\right)=\prod_{v\in V_{s}}f_{Y}\left(y_{sv}\;|\;\tau \left(t_{sv}\right)\right)=\prod_{v\in V_{s}}\prod_{b\in B_{sv}}f_{Y^{b}}\left(y_{sv}^{b}\;|\;\tau \left(t_{sv}\right)\right). \end{array}$$

whereat all biomarker observations are assumed to be independent of each other.

Two different parameterisations of *τ* are regarded. The most simple way is given by the *translational time warp*

$$\begin{array}{c} \tau \left(p^{0};t\right):=p^{0}+t. \end{array}$$ (5)

Here, the *disease progress* (DP) $p^{0}\in R$ is a simple offset that indicates how far the subject is progressed in the course of the disease at the time point of the first visit, measured in days relative to the point of conversion from MCI to AD. However, this simple model cannot accommodate for different rates of progression, which are known to exist between subjects [4]. If |*V*_*s*_|>1, the extended *affine time warp* definition

$$\begin{array}{c} \tau \left(p^{0},r;t\right):=p^{0}+rt \end{array}$$ (6)

can be employed, where $r\in R^{+}$ is a scaling factor indicating the *disease progression rate* (DPR). The optimal values ${\hat{p}}_{s}^{0}$ and ${\hat{r}}_{s}$ for DP and DPR are then determined by maximising Eq (4) over all *p*^0^ and *r*, meaning ${\hat{\tau }}_{s}\left(\cdot \right)\;\hat{=}\;\tau \left({\hat{p}}_{s}^{0},\hat{r};\cdot \right)$. The definition of other, more complex time warps is possible but not regarded in this work. In the current implementation, Eq (4) is solved with a simple exhaustive search.

## Materials

To evaluate the presented methods for model training and progress estimation, synthetic as well as real data is considered.

### Synthetic data

Artificial progression models ${\tilde{M}}^{b}\left(p\right)$ are defined to serve as ground truth in the experiments on synthetic data. Using such models, biomarker values can be randomly sampled on *P* according to their PDFs. Based on these samples, progression models $M^{b}\left(p\right)$ are learned and then compared to the underlying model ${\tilde{M}}^{b}\left(p\right)$.

The synthetic models used in this work are defined by a median trajectory $y_{0.5}^{b}\left(p\right)$ (for example, a linear, exponential or sigmoidal model) and a density function to account for intra- and inter-subject variations. ${\tilde{M}}^{b}\left(p\right)$ are defined loosely based on clinical publications by Henneman et al. [33] and Coley et al. [34]. However, it is to be noted that the intention is not to optimally describe realistic progression models, but rather to cover different density functions that could similarly be observed “in the wild”. With this in mind, the synthetic models analysed in this work represent the following three biomarkers:

- *Hippocampal volume (HV)*: In [33], a mean hippocampal volume of 3,693(±572)mm^3^ and an atrophy rate of 3.5% per year is reported for a pool of 142 subjects. Based on this data, a median trajectory is defined as $y_{0.5}^{\text{HV}}\left(p\right)=3693\cdot 0.965^{p/365}$. The underlying PDF is assumed to be Gaussian, such that ${\tilde{f}}_{Y^{\text{HV}}}\left(y|p\right):=\phi \left(p,y_{0.5}^{\text{HV}}\left(p\right),572\right)$, where *ϕ*(*p*, *μ*^HV^(*p*), *σ*^HV^) denotes a normal distribution with time-varying mean (and median) *μ*^HV^(*p*) and fixed standard deviation *σ*^HV^.
- *CDR-SB*: Coley et al. [34] divide subjects in two groups with a Clinical Dementia Rating (CDR) of 0.5 and 1-2, respectively. For these, CDR-SB (Clinical Dementia Rating: Sum of Boxes) scores of 3.2(±0.9) and 7.9(±2.6) are reported. For the definition of a synthetic model, the disease progression is arbitrarily assumed to equal -1095 (3 years before conversion) for the first group and 1095 (3 years after conversion) for the latter. An exponential model (see Table 1) is defined for the median trajectory such that $y_{0.5}^{\text{CDR-SB}}\left(-1095\right)=3.2$ and $y_{0.5}^{\text{CDR-SB}}\left(1095\right)=7.9$. Equally, a time-dependent standard variation *σ*^CDR-SB^(*p*) is interpolated by an exponential function. Since the CDR-SB is always positive, a Gaussian distribution of the values is unlikely and a gamma distribution is chosen instead: ${\tilde{f}}_{Y^{\text{CDR-SB}}}\left(y|p\right):=\Gamma \left(p,k\left(p\right),\theta \left(p\right)\right)$. Shape and scale parameters *k*(*p*) and *θ*(*p*) are chosen such that median and standard deviation of the PDF equal $y_{0.5}^{\text{CDR-SB}}\left(p\right)$ and *σ*^CDR-SB^(*p*).
- *MMSE*: For the MMSE score, values of 23.2(±2.6) for the CDR 0.5 group and 18, 4(±4.0) for the CDR 1-2 group are reported [34]. Based on these values, the model is defined using a negative Gamma distribution, such that values are bounded at a MMSE score of 30.

**Fig 2:**
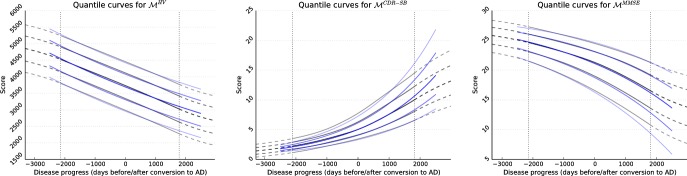
Illustration of the quantile functions of the synthetic models in blue. In grey, models reconstructed from *n*_smp_ = 1000 random samples are shown.

The definitions are detailed in Table 1 and the resulting synthetic density functions visualised in Fig 2.

**Table 1:** Overview on the synthetic models with *p*′ := *p* + 1095.

Model	Median trajectory $y_{0.5}^{b}\left(p\right)$	Noise model	Sigma *σ*^*b*^(*p*)
${\tilde{M}}^{\text{HV}}$	3693*0.965^*p*/365^	Gaussian	572.0
${\tilde{M}}^{\text{CDR-SB}}$	$3.2\exp(\frac{\log(7.9/3.2)}{6*365}p^{\prime })$	Gamma	$0.9\exp(\frac{\log(2.6/0.9)}{6*365}p^{\prime })$
${\tilde{M}}^{\text{MMSE}}$	$30-6.8\exp(\frac{\log(11.6/6.8)}{6*365}p^{\prime })$	Gamma	$2.6\exp(\frac{\log(4.0/2.6)}{6*365}p^{\prime })$

### Real data

Clinical evaluation is based on data from the Alzheimer’s Disease Neuroimaging Initiative (ADNI). ADNI is a large-scale multi-site study that aims at analysing biomarkers from cognitive tests, blood tests, tests of cerebrospinal fluid, and MRI/PET imaging with regard to their ability to characterise the progression of AD.

For this study, all subjects enrolled in either ADNI1, ADNIGO or ADNI2 are considered (based on the ADNIMERGE data base as of 30/01/2014). Participating subjects are categorised as CN, EMCI, LMCI or AD (according to the NINCDS/ADRDA criteria). Levels of MCI (early or late) are determined using the Wechsler Memory Scale Logical Memory II. For model training, all 248 subjects that converted from MCI to AD during the study are selected. As detailed below, the number of available biomarkers can vary between the training subjects. Data from all available visits (between 2 and 11 visits over 96 months, median 6.0) is used for model training. The test set, in contrast, comprises the non-converting subjects for which all biomarkers are present at baseline (bl), month 12 (m12) and month 24 (m24) visit to obtain consistent results in all experiments. In total, these are 160 (89 male, 71 female) subjects classified as cognitive normal (CN), 91 (50 male, 41 female) EMCI, 96 (64 male, 32 female) LMCI and 95 (49 male, 46 female) AD subjects.

The set *B* of biomarkers considered for the real data comprises cognitive scores and image-based features detailed in the following. An age correction of the biomarker values is performed using a linear regression on the baseline samples of all control subjects.

#### Cognitive scores *B*^cog^

Subjects participating in the ADNI study are asked to perform a number of cognitive tests at each visit and the results are directly used as biomarkers. These tests comprise the Mini—mental State Examination (MMSE), the Alzheimer’s Disease Assessment Scale (ADAS 11 and ADAS 13), the Functional Activities Questionnaire (FAQ), the Clinical Dementia Rating—Sum of Boxes (CDR-SB), and the Rey Auditory Verbal Learning Test (RAVLT). It is to be noted that not every test result is available for each visit, such that the absolute number of available training samples varies between 1458 to 1480 samples from 248 subjects.

#### Volumes of brain structures *B*^vol^

Further, the volumes of 35 distinct brain structures are used as biomarkers. For this, MR scans are first automatically segmented into 134 regions using the whole brain segmentation proposed by Ledig et al. [35], which is based on multi-atlas label propagation with expectation-maximisation based refinement (MALPEM). Brain atlases from the MICCAI 2012 Grand Challenge on Multi-Atlas Labeling (https://masi.vuse.vanderbilt.edu/workshop2012) are employed. The corresponding manual expert segmentations are provided by Neuromorphometrics, Inc. (http://Neuromorphometrics.com) under academic subscription. Here, the 30 atlas segmentations are transformed to an unsegmented scan and fused into a consensus probabilistic segmentation estimate using a local weighting approach. The required nonrigid transformations are calculated using a robust registration method based on multi-level free form deformations [36]. All 134 probabilistic label estimates are subsequently corrected for registration inaccuracies and further refined using image intensity information.

To reduce the total number of models, cortical structures are fused to right and left cortex, resulting in the 35 distinct anatomical regions listed in appendix A2. For procedural reasons, only segmentations for images acquired before 20/11/2013 were available, such that the total number of training samples for each structure is 955 from 247 subjects.

#### Biomarkers derived from manifold learning *B*^ml^

Features obtained from MR images using manifold learning (ML) have been shown to contain valuable information about disease severity and progression [28]. The main idea of ML is to find a meaningful, low-dimensional representation of a high-dimensional feature space, such that similar scans also have similar coordinates in the low-dimensional manifold. This is achieved in three steps. First, the image regions that are most relevant with regard to information about disease progression are automatically learned using sparse regression as in [37]. To compensate for varying intensity values in the images caused by different scanners and acquisition protocols, local binary patterns (LBP) are computed in a 26-connected neighbourhood for voxels within these regions and used as features in the high-dimensional space. The manifold is then learned using Laplacian eigenmaps. The local geometry is determined via a sparse similarity graph, built using the sum of squared differences (SSD) as similarity measure. Connections in the graph are made between the *k* nearest neighbours, with the additional constraint that an instance can only be connected to one instance per subject.

The manifold coordinates are computed for all training subjects with at least 5 visits, which results in 859 samples from 155 subjects available to train the models. The manifold is chosen to have a dimension of *d* = 20, that means 20 features are obtained per subject per visit and denoted with D1 to D20.

## Results

### Synthetic data

Evaluation based on synthetic data follows two main goals: A) to analyse how much the model training based on quantile regression depends on the available data, and B) to demonstrate the principle functionality of the presented approach for progress estimation.

#### Model reconstruction

The main interest of the model reconstruction is to analyse to which degree VGAMs depend on the quantity of available training data. For all experiments, the principle procedure is the following: First, a set of random samples is generated based on the density functions defined by the synthetic models ${\tilde{M}}^{b}$. Then, these samples are used to estimate the underlying model, yielding the reconstructed model $M^{b}$. Reconstruction accuracy is assessed by comparing ${\tilde{M}}^{b}$ to $M^{b}$. As metric, the mean area between the corresponding density functions is regarded:

$$\begin{array}{c} {\text{AREA}}^{b}:=\frac{1}{\left|P\right|}\int_{P}\int_{y}\left|f_{Y^{b}}\left(y|p\right)-{\tilde{f}}_{Y^{b}}\left(y|p\right)\right|\;dy\;dp \end{array}$$

To simulate the properties of the real data, the distribution of the samples on the domain *P* requires special consideration. In particular, three sampling strategies are pursued:

- Uniform sampling: The actual data structure is ignored and each sample is regarded independently from the others, leading to a uniform sampling over *P*.
- Triangular sampling: For the model training based on real data, longitudinal time series are aligned according to the point of conversion, which can be between any of the considered visits. As a result, the density of samples decreases with the distance to *p* = 0. This effect can be approximated by a triangular distribution of the samples over *P*. Note that by the ADNI protocol, subjects are only followed for a certain period of time after AD diagnosis and the distributions are therefore not symmetric at *p* = 0. Still, each sample is regarded independently from the others, which means that the variation of the PDF *f*_*Y*^*b*^_ is solely explained by noise in the measurements.
- Longitudinal sampling: In this sampling approach, the variation in the density functions is assumed to only account for inter-subject variations. That means, only the first sample in a series of *n*_*V*_ = 6 samples per subject is randomly distributed according to the noise model. The following time points are sampled along the same quantile curve as the first sample with *q* = *q*^*b*^(*y*_*s*1_, *p*_*s*1_).

**Fig 3:**
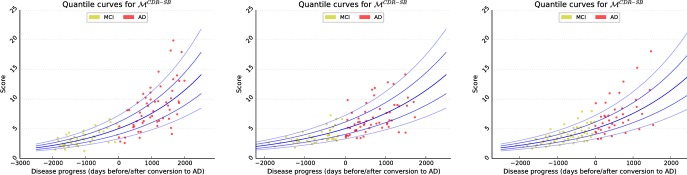
Illustration of 100 random samples generated using uniform (left), triangular (center) and longitudinal (right) sampling. The underlying model ${\tilde{M}}^{\text{CDR-SB}}$ is shown in blue.

The three sampling strategies are visualised in Fig 3. Real data can be assumed to lie somewhere between triangular and longitudinal sampling, as the variations are explained by a mixture of inter-subject variability and noise.

With the real data pool in mind, four properties of the training set are in the focus of the conducted experiments: 1) the number of samples, 2) the distribution of the samples over *P*, 3) inaccuracies in the temporal alignment, and 4) variances in the progression rates of the subjects. Further, the approach is compared to a competing model.

**Fig 4:**
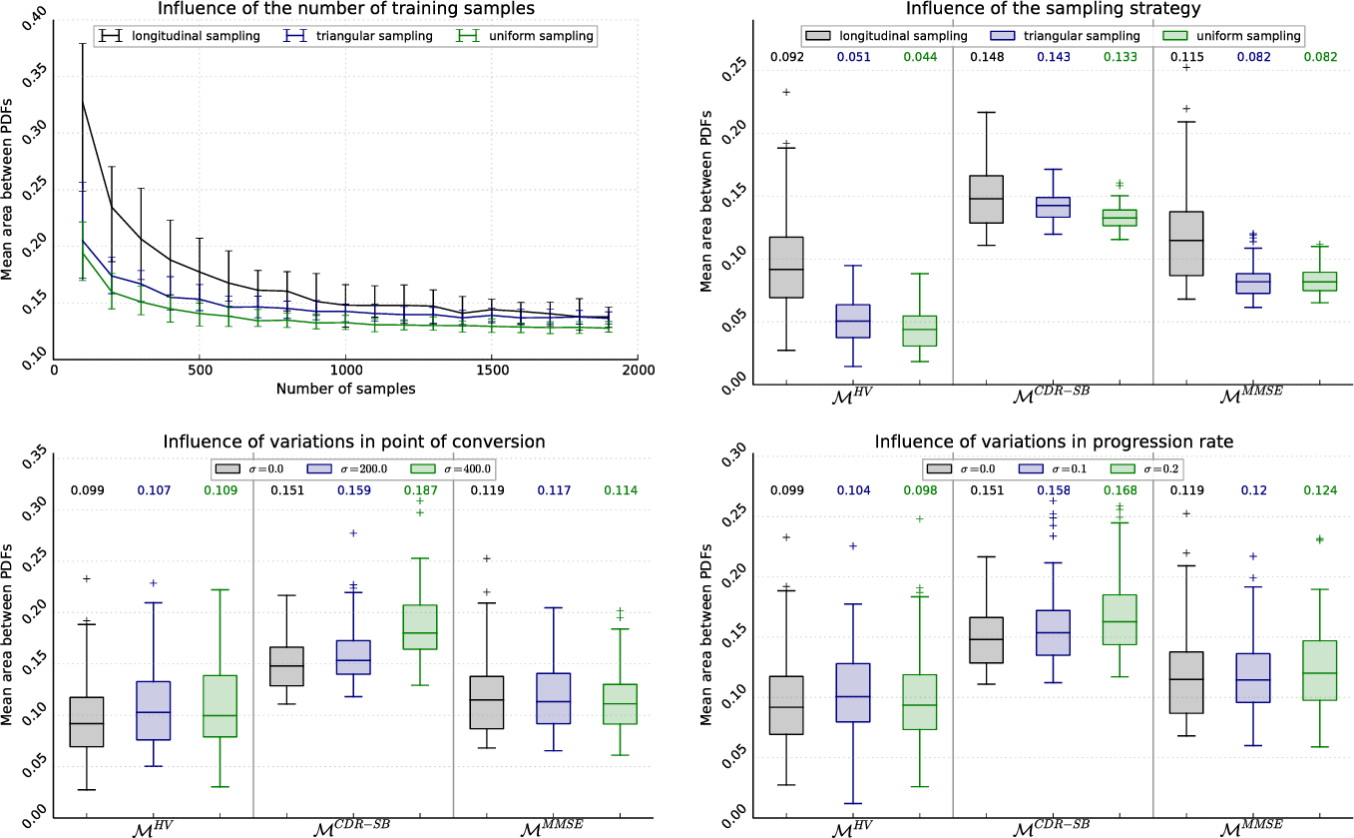
To measure the influence of the data pool on the model training, the sensitivity of quantile regression using VGAMs to different properties of the sampling set is analysed. The graphs show the mean reconstruction error after 100 cycles of random sample generation and model training. The numbers above the boxes indicate the median error. (A) Sensitivity to the number of samples. (B) Sensitivity to the sampling strategy. (C) Robustness against temporal misalignment. (D) Sensitivity to different progression rates.

- Sensitivity to the number of samples: To analyse the influence of the number of samples on the model training, the reconstruction procedure is executed with each sampling strategy for *n*_smp_ = 100, 200, …, 1900 samples. The influence of randomness is reduced by running each experiment *n*_runs_ = 100 times. The mean errors and the 25th and 75th percentiles are—exemplarily for $M^{\text{CDR-SB}}\left(p\right)$—plotted in Fig 4A. It is observed that the estimation drastically improves until about 500 samples are reached, then slowly converges and reaches a stable level at around 1500 samples. Equally, the variation due to randomly chosen samples decreases. Regardless of the number of samples, uniform sampling performs slightly better than the triangular sampling, against which the longitudinal sampling converges at around 1500 samples.
- Sensitivity to the sampling strategy: To better assess the influence of the sampling strategy, the results of the first experiment are visualised for all models in Fig 4B. Here, models are learned based on 1000 samples, which approximately corresponds to the number of measurements available for the real data. The trend “longitudinal >> triangular > uniform” persists, especially with regard to the variation. However, the influence of the specific model outweighs this effect, with $M^{\text{CDR-SB}}\left(p\right)$ being the worst reconstructed model and $M^{\text{HV}}\left(p\right)$ the best.
- Robustness against misalignment: In this work, the time of conversion $t_{s}^{0}$ is assumed to be in the center between the last MCI and the first AD diagnosis. However, in real data it can be everywhere between these two visits. Moreover, the diagnoses can be erroneous (or, more precisely, the threshold at which a subject is classified as AD inconsistent), which entails a more severe misalignment. To analyse the influence of this, the progress *p*_*sv*_ associated with the samples (longitudinal sampling) is disturbed before model generation by adding artificial noise, meaning *p*_*sv*_ ← *δ*_*s*_ + *p*_*sv*_, where *δ*_*s*_ is sampled from a Gaussian distribution with *μ* = 0 and *σ* ∈ [0, 200, 400]. The results are given in Fig 4C. While the influence of noise with *σ* = 200 is only marginal, a decline in accuracy can be observed for *σ* = 400. Still, the difference between the models is much higher than the influence of noise.
- Sensitivity to different progression rates: Lastly, subjects are all assumed to progress with the same rate in the model estimation approach, while in reality considerable differences in the progression speed can be observed [3]. Therefore, similarly to the preceding experiment, noise is added on the samples using *p*_*sv*_ ← *δ*_*s*_ ⋅ *p*_*sv*_. Here *δ*_*s*_ is Gauss-distributed with *μ* = 1 and *σ* ∈ [0.0, 0.1, 0.2]. The results depicted in Fig 4D show no considerable difference between the different noise levels, for neither of the biomarkers.
- Comparison to competing model: In addition, the estimated models are compared to the approach presented by Donohue et al. [22]. This approach was chosen for several reasons. First, both methods share the property that no assumptions on the shapes of trajectories are made besides smoothness (and, in case of [22], monotony). Second, an open source implementation of Donohue’s model is available (http://mdonohue.bitbucket.org/grace/), which allows a comparison based on the same set of samples. Finally, the approach stands exemplarily for methods that do not require a diagnosis for pre-alignment and are therefore not susceptible to mis-diagnosis.
  For this reason, models have been built for the data used in Fig 4C (100 runs, 1000 samples, longitudinal sampling, *σ* ∈ [0, 400]). As only the mean trajectories and not the quantile curves are estimated by [22], the average difference between the mean trajectories is regarded as metric. Without relying on the clinical diagnosis as input data, Donohue’s approach allows a good reconstruction of the three models $M^{\text{HV}}$, $M^{\text{CDR-SB}}$ and $M^{\text{MMSE}}$ with mean differences of 60.6, 0.59 and 0.77 units. Assuming knowledge of the correct clinical diagnosis (i.e., *σ* = 0), VGAM’s provides a more precise reconstruction with mean errors of 42.7, 0.146 and 0.264 units. Simulating mis-diagnosis by introducing an error on the temporal alignment, errors increase (42.5, 0.181, and 0.293 units for *σ* = 400) but are still considerably smaller than for Donohue’s model.

#### Progress estimation

The accuracy of the model reconstruction regarded in the preceding section does not allow any conclusion about the suitability of the models for estimating the disease progression (even though it obviously is a prerequisite): A perfectly reconstructed model that does not change with *p* contains no information regarding the DP. Therefore, the focus of this section is on quantifying the accuracy of progress estimation. To this end, *n*_test_ random test samples are generated in the same way as the training samples, using uniform sampling over *P*. These samples are then fitted to the model using Eq (4) to estimate the disease progress that corresponds to the sample. The mean error between the estimated progress and the progress assumed during sampling is regarded as metric.

**Fig 5:**
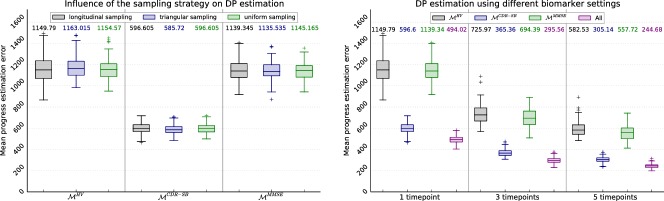
To illustrate the functionality of progress estimation for synthetic data, the mean estimation errors are computed based on a set of *n*_test_ = 100 randomly generated test samples. The figures show the mean errors for *n*_runs_ = 100 runs of the experiments. The models correspond to the models analysed in Fig 4B. (A) Sensitivity to the sampling strategy. (B) Influence of additional data from more visits and multiple biomarkers.

- Correlation between model reconstruction and estimation accuracy: First, the disease progress is estimated for *n*_test_ = 100 individual single-biomarker samples with a search space of [−3500, 2500] (which considerably exceeds the domain of the model). For this, the models learned for Fig 4B are employed. In each run, a new set of test samples is generated to compensate for the effect of randomness. The mean errors are illustrated in Fig 5A. While the differences between the models is quite striking with $M^{\text{CDR-SB}}\left(p\right)$ being superior to the other models, the influence of the sampling strategy is negligible.
- Estimation with multiple biomarkers: In all preceding experiments, single biomarker values were fitted to the corresponding models. Here, the influence of multiple measurements is analysed, which can stem either from multiple visits or the simultaneous consideration of multiple biomarkers. Therefore, the samples of the individual biomarkers are merged and fitted to the multi-biomarker model by regarding the joint probability. Moreover, the experiments are repeated with samples from 3 and 5 visits, while using longitudinal sampling for sample generation. The results are displayed in Fig 5B. As can be seen, the estimation accuracy greatly improves with the number of biomarkers and visits.

### Real data

Complementary to the experiments on synthetic data, this section is to evaluate the presented approach based on biomarkers derived from clinical image data. First, progression models are trained for all considered biomarkers based on the ADNI data. These models are then employed to estimate DP and DPR for all test subjects. Since the ground truth is unknown, the meaningfulness of the estimated values is indirectly assessed by employing them in three exemplary applications: progress estimation for classification, construction of a 4D atlas of disease progression, and the prediction of biomarker values.

#### Model training

**Fig 6:**
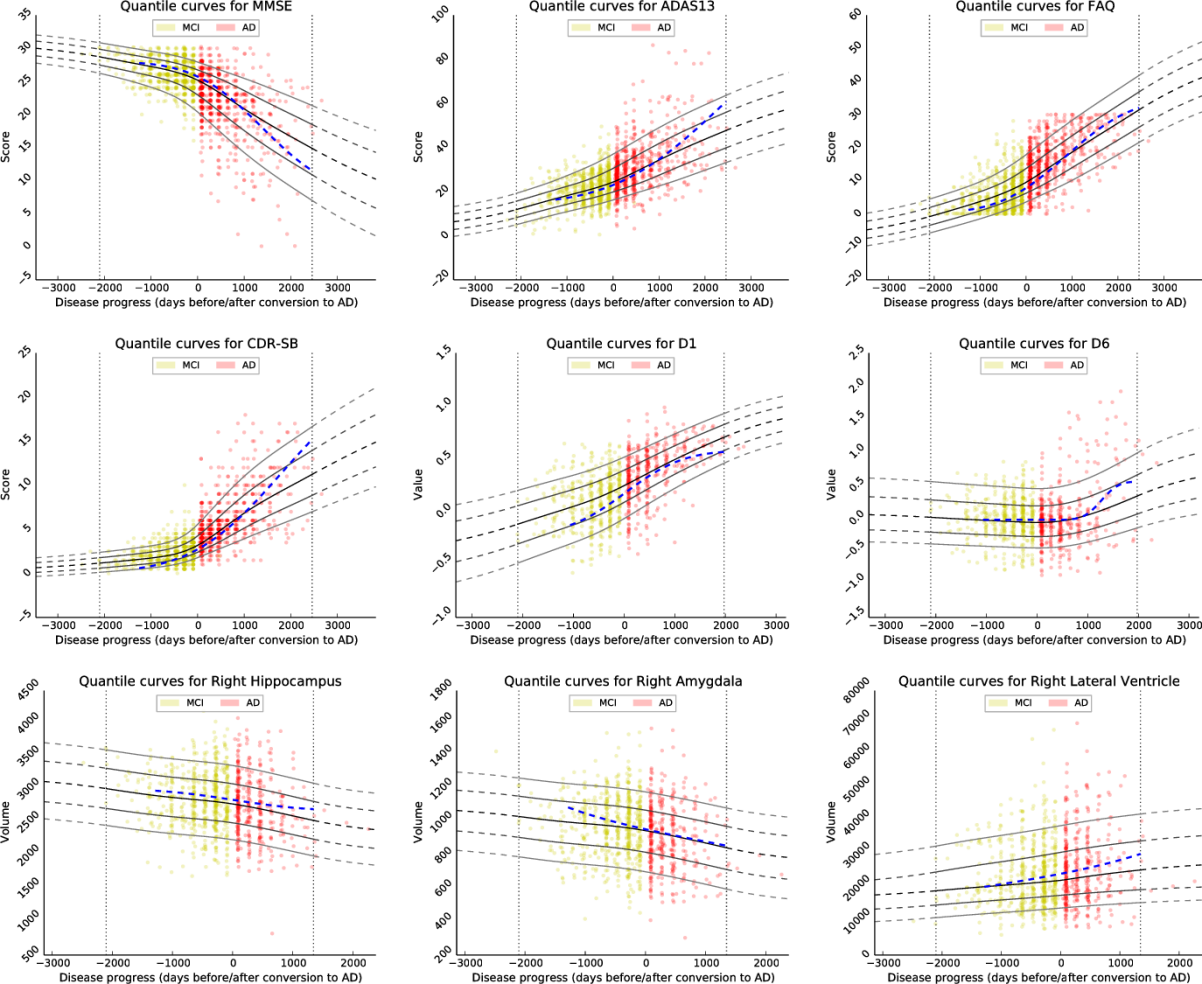
Examples for progression models of several biomarkers learned based on the ADNI data base. Visualised biomarkers are: Mini—mental State Examination (MMSE), the Alzheimer’s Disease Assessment Scale, 2013 (ADAS13), the Clinical Dementia Rating—Sum of Boxes (CDR-SB), the Functional Activities Questionnaire (FAQ), volumes of right hippocampus, amygdala and lateral ventricle, as well as the first and sixth manifold coordinates D1 and D6. In blue, models generated using the approach of Donohue et al. are shown for comparison [22].

Progression models are trained for all 60 regarded biomarkers. The (single-threaded) quantile regression using VGAM takes approximately 15 seconds computation time per biomarker on a standard desktop computer. Exemplary results are visualised in Fig 6. Since the ground truth of the models is unknown, the focus is on quantifying how well the trained models are suited to discriminate between different stages of disease. For this purpose, a model should optimally have a large slope and a small variance. Let $y_{-}^{b}:=\min_{p\in P}y_{0.5}^{b}\left(p\right)$ and $y_{+}^{b}:=\max_{p\in P}y_{0.5}^{b}\left(p\right)$ be the minimum and maximum values of the median trajectory of the model. The interval $Y_{0.5}:=\left[y_{-}^{b},y_{+}^{b}\right]$ then indicates the range of values for which the highest probability lies within *P*. A large range indicates that the peaks of the density functions *f*_*Y*_ are far apart from each other and thus distinctive. Further, the *q*-quantiles $q^{b}\left(y_{-}^{b},p\right)$ and $q^{b}\left(y_{+}^{b},p\right)$ that correspond to the values $y_{-}^{b}$ and $y_{+}^{b}$ are regarded. The distance $q_{+}^{b}\left(p\right)-q_{-}^{b}\left(p\right)$ indicates the ratio of the samples with a value that lies within *Y*_0.5_. A high ratio implies a small variation of the density functions an thus a discriminative model. The total *discriminativeness* of the model is quantified as:

$$\begin{array}{c} {\text{DISC}}^{b}:=\frac{1}{\left|P\right|}\int_{P}q^{b}\left(y_{+}^{b},p\right)-q^{b}\left(y_{-}^{b},p\right)dp. \end{array}$$

In Table 2, the 10 most discriminative models are listed together with the metric DISC^*b*^.

**Table 2:** Ranking of the 10 most discriminative progression models. D1 and D2 denote the first two ML coordinates.

Rank	Biomarker *b*	DISC^*b*^	Rank	Biomarker *b*	DISC^*b*^
1.	FAQ	0.776	6.	ADAS11	0.697
2.	CDRSB	0.764	7.	MMSE	0.651
3.	D1	0.747	8.	Left Amygdala	0.480
4.	ADAS13	0.714	9.	Right Hippocampus	0.431
5.	D2	0.714	10.	Left Hippocampus	0.404

Analogous to the experiments on synthetic data, the estimated models are compared to the approach presented by Donohue et al. [22] using same training data. For transferring the models to the same coordinate system, a temporal offset is optimally determined by minimising the model difference (here: 300 days). The resulting models are visualised in Fig 6.

#### Progress estimation

**Fig 7:**
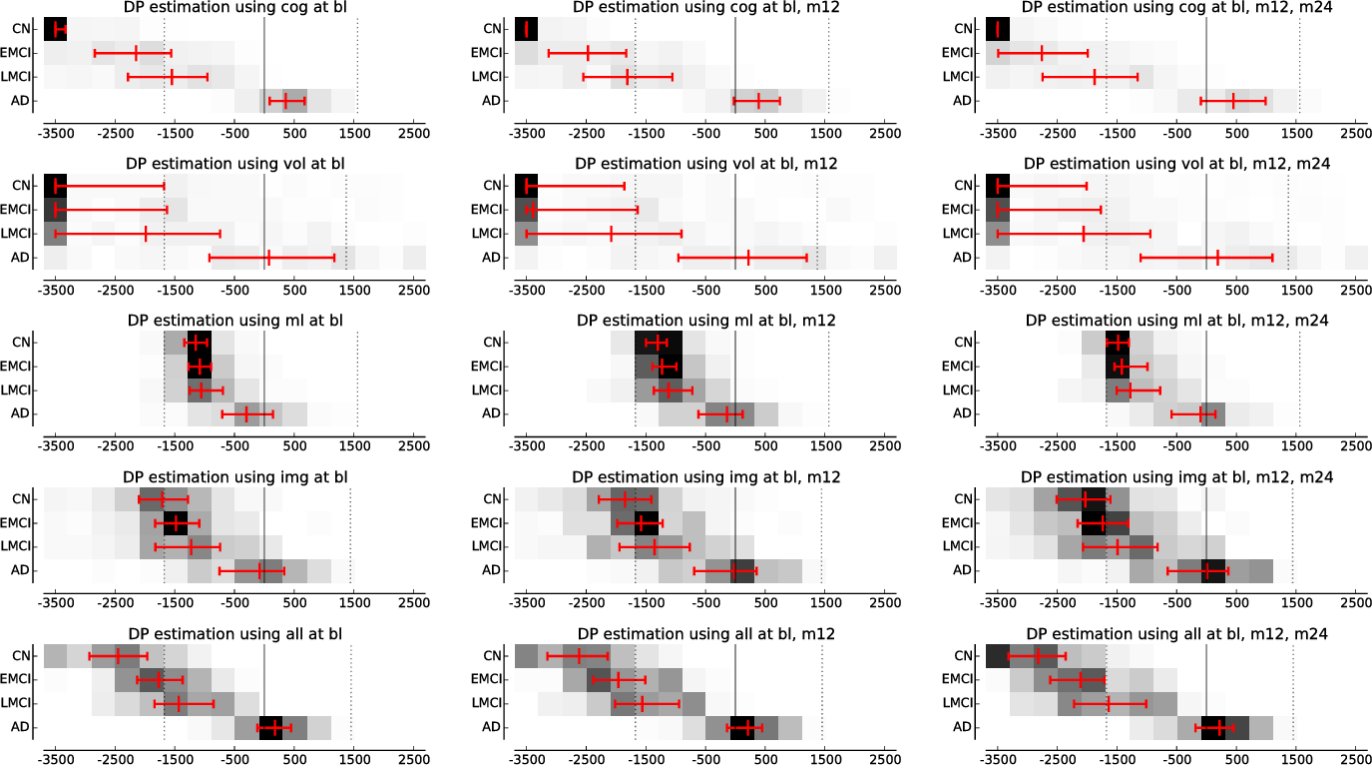
Visualisation of the disease progress (DP) estimated with different biomarkers. The x-axes show the disease progress in days before/after the conversion to AD. In the three columns, data from one, two and three visits is employed. The rows show results based on the different biomarker sets. The red bars indicate the median and 25th/75th percentile of the estimated DPS.

The disease progress of all test subjects is estimated as presented. Since the ground truth is unknown, plausibility of the estimated DPs is evaluated with regard to their ability to differentiate between the diagnoses, that means to what extend an ordering of CN < EMCI < LMCI < AD is achieved. To this end, ${\hat{p}}_{s}^{0}$ is estimated on the search space [−3500, 2500] for all CN, EMCI, LMCI and AD subjects in the test set using several biomarker configurations. On the one hand, different sets of biomarkers *B*^est^ are considered for estimation: *B*^cog^, *B*^vol^, *B*^ml^, the imaging-based biomarkers *B*^img^ := *B*^vol^ ∪ *B*^ml^, and all biomarkers united *B*^all^ := *B*^cog^ ∪ *B*^img^. On the other hand, biomarkers from one (baseline), two (baseline and m12) and three (baseline, m12 and m24) visits *V*^est^ are employed. The distribution of the estimated DPs depending on the diagnosis is visualised in Fig 7.

#### Application: Classification based on disease progress

One of the main research topics of image-based analysis of Alzheimer’s disease is the classification of subjects according to their diagnosis based on structural MRI scans. This is underlined by a classification challenge held in the course of MICCAI 2014 conference, at which 15 international groups participated with total of with 29 algorithms [38].

Therefore, the estimated DPs are used for a two-class classification between CN and AD, CN and MCI, MCI and AD, as well as EMCI and LMCI. With only one feature, classification breaks down to finding an optimal threshold *p*^thresh^ that separates one group from the other. For this test, the cognitive scores are excluded because the diagnosis is made largely based on the CDR, such that including them biases the classification (e.g., classification accuracy reaches 1.0 for CN vs. AD and 0.96 for MCI vs. AD using cog). The average results of 100 runs of a 10-fold cross-validation are shown in Table 3.

**Table 3:** Results for a classification based on the DP as single feature, which is estimated using image-based biomarkers (see Fig 7). The numbers indicate the mean accuracy (ACC), sensitivity (SENS) and specificity (SPEC) after 100 runs of a 10-fold cross-validation.

Biomarkers	Visits	CN vs. AD			CN vs. MCI			MCI vs. AD			EMCI vs. LMCI		
*B*^est^	*V*^est^	ACC	SENS	SPEC	ACC	SENS	SPEC	ACC	SENS	SPEC	ACC	SENS	SPEC
*B*^vol^	{bl}	0.78	0.81	0.77	0.66	0.64	0.70	0.66	0.64	0.67	0.51	0.42	0.74
	{bl, m12}	0.80	0.82	0.79	0.66	0.66	0.67	0.68	0.66	0.68	0.52	0.38	0.87
	{bl, m12, m24}	0.81	0.78	0.82	0.66	0.65	0.67	0.68	0.68	0.68	0.54	0.40	0.87
*B*^ml^	{bl}	0.84	0.82	0.85	**0.69**	0.66	0.72	0.73	0.72	0.73	0.53	0.43	0.79
	{bl, m12}	0.87	0.84	0.88	0.66	0.61	0.76	**0.74**	0.69	0.76	0.57	0.51	0.74
	{bl, m12, m24}	0.88	0.87	0.89	0.65	0.56	0.79	0.71	0.69	0.72	0.56	0.49	0.73
*B*^img^	{bl}	0.84	0.88	0.83	0.68	0.64	0.74	0.69	0.74	0.67	0.54	0.45	0.75
	{bl, m12}	0.87	0.86	0.87	0.68	0.63	0.75	0.71	0.80	0.69	0.57	0.48	0.79
	{bl, m12, m24}	**0.89**	0.89	0.89	0.67	0.60	0.79	0.70	0.80	0.68	**0.58**	0.50	0.78

#### Application: 4D atlas construction

Anatomical shape and appearance atlases play an important role in the representation of populations of subjects and in the quantitative analysis of variations between them. Spatio-temporal (4D) atlases additionally provide information about physiological processes and have been applied in particular to study brain development in paediatrics [39], neonatology [40], and in ageing [41]. In these applications, the subject’s age at the time of image acquisition is generally considered as the temporal dimension. Further, powerful techniques exist to analyse anatomical changes over time caused by ageing or AD. These include (but are not limited to) statistics on temporal deformations [27], multivariate regression to explore relations between shape and clinical response [42], or regional flux analysis using Helmholtz decomposition of stationary velocity fields [43]. Lorenzi et al. further present an approach to disentangle normal ageing from pathological changes. Such methods allow to gain valuable information about characteristics of disease-related morphological changes [44].

In this section, however, the main goal is to visually assess validity of the estimated DPs by constructing a spatio-temporal atlas of the disease progress and identifying deformations typical for AD. To this end, the bias-free atlas construction proposed by Serag et al. [45] is employed, using DP instead of age to determine the temporal variable. First, the DP is estimated for all MCI and AD subjects in the training set using all biomarkers *B*^est^ = *B*^all^ at three timepoints *V*^est^ = {1, 2, 3} (here, baseline, m12 and m24), which coincides with the last experiment in Fig 7. The baseline scans are then ordered according to their DP (instead of their age) and adaptive kernel regression is employed to construct the atlas. To compute the mean geometry at a specific disease progress *p*, only subjects with a DP close to *p* are considered and their weighted average is computed as detailed in [45] (with the only difference that a symmetric diffeomorphic registration approach [46] is employed for an intrinsic availability of the inverse transformation).

**Fig 8:**
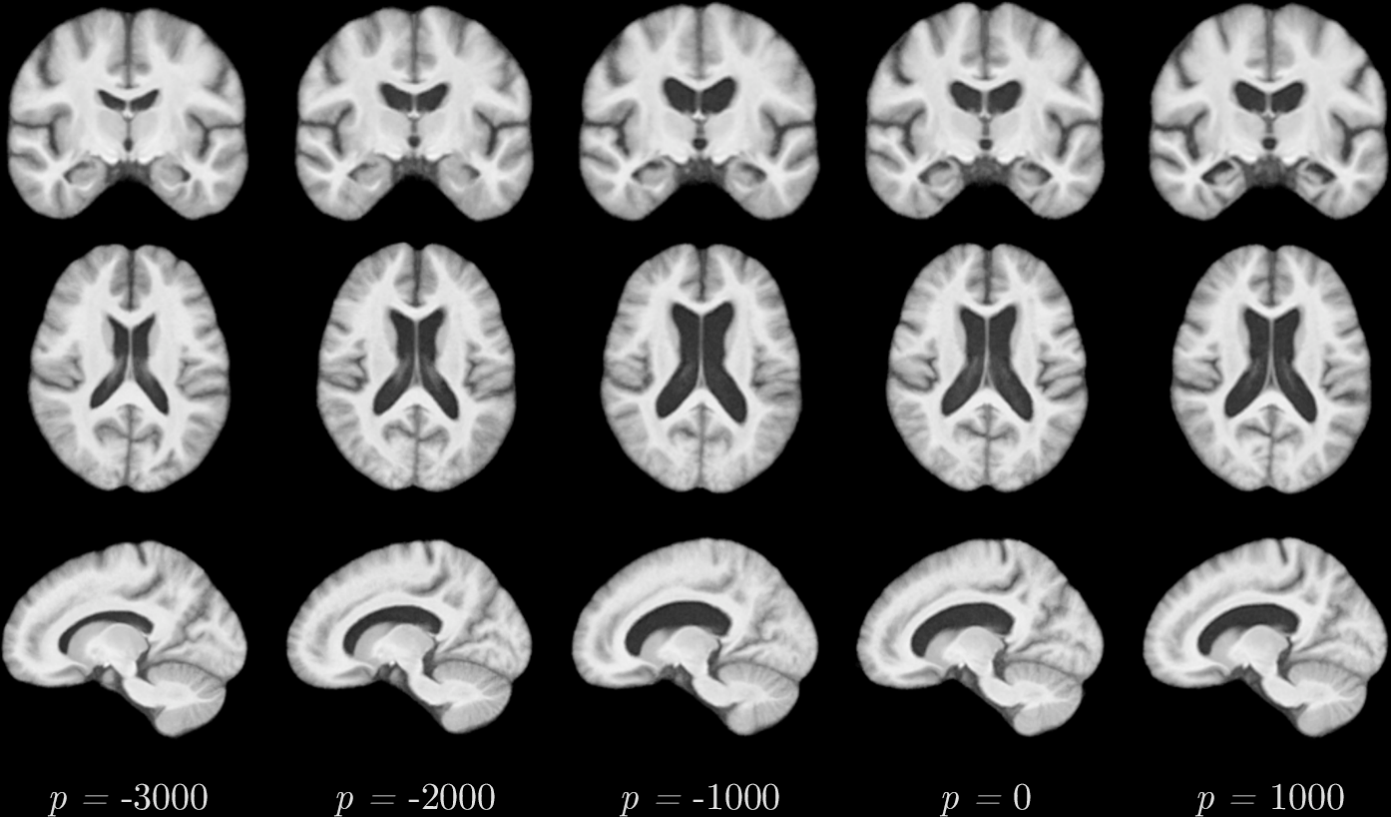
The generated 4D atlas depicting the the progression of Alzheimer’s disease, disentangled from the normal ageing of the subjects.

The constructed 4D atlas of the brain degeneration is visualised in Fig 8 for *p* ∈ [−3000, −2000, −1000, 0, 1000] days.

#### Application: Prediction of biomarker values

As initially motivated, an important requirement for clinicians is the prediction of the future course of disease, in particular the cognitive and functional decline. Here, the development of biomarker values is predicted based on the approach for DP and DPR estimation. In detail, the values $y_{sv}^{b}$ of a biomarker *b* are assumed to be known for subject *s* for three timepoints *t*_*sv*_, with *v* ∈ *V*^est^ = {1, 2, 3}. Based on these samples, the value $y_{s4}^{b}$ at *t*_*s*4_ is predicted. To do so, three prediction approaches are compared:

- *Naive prediction*: A naive prediction is employed as reference approach. Here, a linear model $y_{s}^{b}\left(t\right):=\bar{m}t+a_{s}$ is fitted to the three observations, where the slope $\bar{m}$ is the average change of the biomarker values in the course of one year, which is estimated on all MCI and AD subjects. It is then $a_{s}=-\frac{1}{3}\sum_{v}\left(\bar{m}t_{sv}-y_{sv}^{b}\right)$. The predicted biomarker value is given by ${\tilde{y}}_{s4}^{b}=y_{s}^{b}\left(t_{s4}\right)$.
- *DP-based prediction*: For a model-based prediction, the DP is estimated for *s*—corresponding to the last column in Fig 7—using all three visits and a biomarker set *B*^est^. Note that *b* might or might not be included in *B*^est^. From a practical point of view, it makes sense to always consider *b* if values are available, however, it is not done here to be able to correlate the prediction results with the progress estimation shown in Fig 7. Based on the estimated processes $p_{sv}=\tau \left({\hat{p}}_{s}^{0};t_{sv}\right)$, the mean quantile curve
  $$\begin{array}{c} {\bar{q}}_{s}=\frac{1}{3}\sum_{v=1}^{3}q^{b}\left(y_{sv}^{b},p_{sv}\right) \end{array}$$
  is then determined. The value $y_{s4}^{b}$ is assumed to lie on the mean quantile at *t*_*s*4_, that means ${\tilde{y}}_{s4}^{b}=y_{{\bar{q}}_{s}}^{b}\left(p_{s4}\right)$
- *DP/DPR-based prediction*: To also account for the progression rate of *s*, the same approach is repeated with the affine time warp definition Eq (6), that means $p_{sv}=\tau \left({\hat{p}}_{s}^{0},{\hat{r}}_{s};t_{sv}\right)$

**Fig 9:**
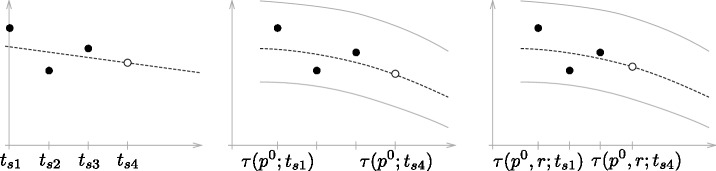
Concept of naive (left), DP- (center) and DP/DPR-based (right) prediction of biomarker values.

**Fig 10:**
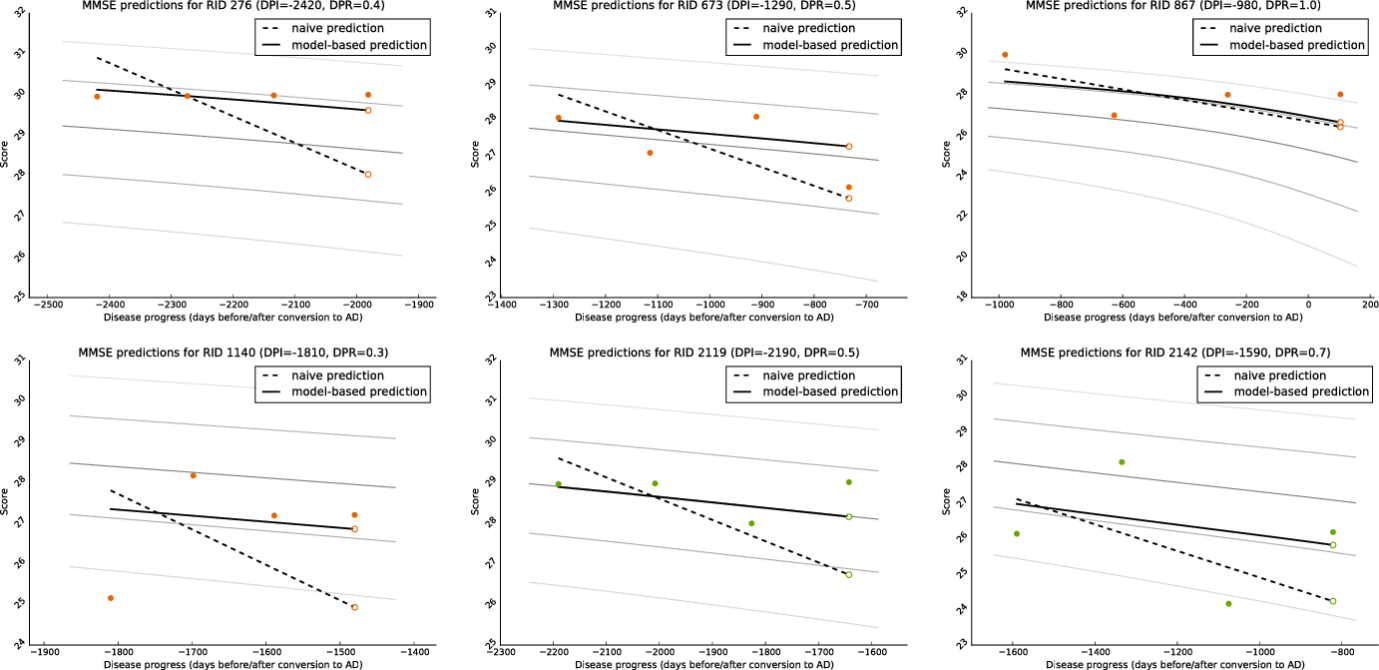
Illustration of observed (filled circles) and predicted (outlined circles) biomarker values for six randomly picked and representative subjects. The slope of the naive linear prediction approach is visualised with a dashed line with $y_{s}^{b}\left(t\right)$ at the end. In a solid line, the quantile curve ${\bar{q}}_{s}$ is shown. The fitted progression model is shown in light grey.

**Fig 11:**
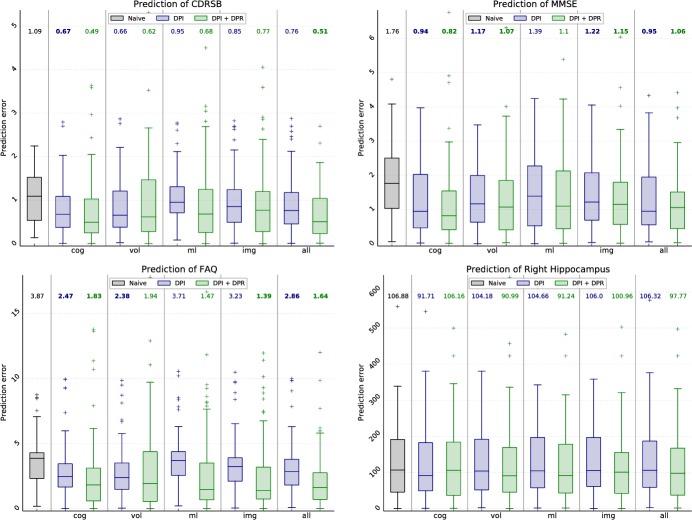
Results for the prediction of future biomarker values for four different biomarkers (tree cognitive scores and the hippocampal volume). The prediction of the value at m36 is based on bl, m12 and m24 visits, using the ADNI data. Bold median values indicate a statistically significant improvement over the naive approach (*p* < 0.01).

Fig 9, shows sketches to illustrate these three approaches. Further, some exemplary results are given in Fig 10. The prediction errors $|y_{s4}^{b}-{\tilde{y}}_{s4}^{b}|$ for the CDR-SB and MMSE (indicating cognitive decline), FAQ (functional decline) and the volume of the right hippocampus (to show the versatility of the approach) using different biomarker sets are given in Fig 11. These results are based on all AD and MCI subjects for which $y_{s4}^{b}$ is additionally available for evaluation, resulting in 77 subjects.

## Discussion

### Synthetic data

The experiments on synthetic data demonstrate that VGAMs are suitable for reconstructing the probability density functions of a progression model, given a sufficient number of samples. In this context, *n*_smp_ ≈ 1000 appears to be adequate to compensate for the effect of the longitudinal sampling. However, more samples could decrease the impact of randomness. It is further observed that the reconstruction error increases with the distance from *p* = 0 due to the lower density of samples. The dependence on long biomarker sequences—this means, sequences of samples acquired during as many visits as possible—is therefore given for two reasons: On the one hand, to extend the interval *P* on which the model is learned and on the other hand, to increase the density of samples at the end points and thereby decrease the reconstruction error in these regions. In practice, this requirement limits the applicability of the model learning procedure to rather large data sets like ADNI.

In the experiments, the influence of temporal misalignment and in particular different progression rates of the training subjects was small and negligible. This was especially apparent when analysing the estimation error, because the disciminativeness of the specific model clearly outweighs the impact of the reconstruction error. For example, while a considerable influence of the sampling strategy on the reconstruction is apparent in Fig 4B, no considerable difference of the estimated DPs is observed in Fig 5A.

In contrast to the model presented by Donohue et al. [22], the presented approach relies on a clinical diagnosis for pre-alignment. The comparison of both methods shows that this additional information can be exploited by VGAM’s for a more precise model reconstruction. While this also implies that the approach is susceptible for mis-diagnosis, the increase in accuracy outweighed the errors introduced by a reasonable degree of misalignment in the experiments.

Adding further information in the form of more biomarker measurements or data from multiple visits significantly increases the accuracy of progress estimation. As shown in Fig 5B, even the hardly discriminative models for MMSE and HV contribute to the overall accuracy when combined with the CDR-SB.

### Real data

Turning to the real data, the models learned using quantile regression and illustrated in Fig 6 produce plausible biomarker trajectories. Cognitive scores and volumetric biomarkers exhibit sigmoidal or near-linear shapes, confirming the observations made, for example, in [11] and [22]. The generated models are similar to the models of Donohue et al.; the mean error is between 0.5% (right cerebral wight matter) and 7.7% (CDR-SB), normalised with the maximal absolute value of the mean trajectory. In some cases, the sigmoidal-shaped models of Donohue et al. exhibit unrealistically large slopes at the boundary of the model domain (cf. right amygdala) or are not able to capture the shape of ml-based biomarkers (cf. D6). However, missing a ground truth, a quantitative comparison of the models’ correctness cannot be given.

In the biomarker ranking shown in Table 2, all five cognitive scores are amongst the most discriminative biomarkers and only the first two manifold coordinates perform similarly well. This finding is reasonable because cognitive scores exhibit a smaller inter-subject variability than, for example, the absolute volumes of brain structures. On the other hand, the good ranking of *B*^cog^ could also point to a potential bias in these models: Since diagnosis in the ADNI protocol is heavily dependent on MMSE and CDR, these scores are implicitly used for temporal alignment of the training subjects. However, due to the fact that MMSE is not significantly better ranked than the other scores (in fact, it is slightly worse), we hypothesise that a potential bias introduced hereto can be assumed to be small.

A further potential bias is introduced by the temporal alignment. Since this step is only based on converters, the models do not represent the whole population if converters progress differently in the disease than non-converters (not only faster), or if certain biological (brain reserve, age) or genetic (APoE-*ϵ*4 presence) factors differ between the two subgroups. While this can be seen as a drawback of the model, constraining the data pool to converters to AD is consistent with the intention to model the progression of Alzheimer’s Disease and therefore assumed to be justified.

A major advantage of the presented approach lies within the fact that all available training samples can be used for learning, even if the total number of samples varies from model to model. This enables an optimal usage of the data (as seen for the synthetic data, more samples entail a better fitting) because, for example, a subject’s CDR-SB score could be used even though no MMSE was acquired. In contrast to that, other common machine learning approaches would be restrained to the set of subjects for which all considered biomarkers are available.

The DPs estimated for the test subjects and shown in Fig 7 show a good class separation. In general, the cognitive scores perform best for distinguishing between the four classes. Regarding the image-based features, *B*^ml^ outperforms the volumetric measurements for MCI/AD separation because these suffer from the large inter-subject variability. Adding more time points slightly improves the results, mainly by enhancing the robustness of the estimate. This can be attributed to the fact that 24 months are—compared to the whole course of disease—a relatively short period of time. In this context, the results for data with a DP not contained in the training set are of particular interest: Using the cognitive and volumetric biomarkers, the majority of CN subjects are correctly clustered at *p* = −3500, that means at the beginning of the search space, even though no control subjects were considered for model learning. Also, early and late MCI subjects have distinct means at *p* < −1500, that means in the extrapolated region. This implies that the model extrapolation is well-suited for *B*^cog^ and *B*^vol^, whose trajectories are monotonically increasing or decreasing functions—as propagated, for example, in [18]. However, this does not hold true for the manifold coordinates, which don’t exhibit this “natural” shape. Extending these models to regions outside the training set remains subject of future research.

These findings are supported by the results for image-based classification shown in Table 3. In general, results CN vs. AD classification (accuracy/sensitivity/specificity up to 0.89/0.89/0.89) using the disease progress as a single feature are on par with recent publications like [47] (reported sensitivity/specificity: up to 0.82/0.89) or [48] (reported accuracy/sensitivity/specificity: 0.89/0.93/0.85). Keeping in mind that slightly different subject sets are used and results are therefore not fully comparable, this demonstrates the potential of the DP to capture as much information about the disease state as the underlying biomarkers. Since the model is focussing on the MCI-to-AD phase, MCI vs. AD classification is considerably better than CN vs. MCI classification. This means, class separation suffers from less accurate modelling of early disease progression, which highlights the potential benefit of extending models to the CN-to-MCI phase.

The applicability of the estimated DPs for the construction of a spatio-temporal atlas of the disease progression has been successfully shown. As illustrated in Fig 8, the 4D atlas exhibits the expected patterns as an increasing volume of the ventricles and hippocampal atrophy.

Further, DPs and DPRs have been employed successfully to predict biomarker values, in particular the expected cognitive decline of a subject. Model-based prediction outperforms the naive approach for all biomarkers considered with only one exception (prediction of the hippocampal volume based on *B*^vol^). Considering the progression rate further improves the prediction. This holds true for all biomarkers used for DP/DPR estimation, however, the cognitive scores generally perform best. An interesting finding is that the manifold coordinates provide the best DP (and DPR) estimation to predict the hippocampal volume. This is intriguing because due to the feature selection, the manifold is learned on a region around the hippocampus and it can be assumed that specific manifolds could be learned for certain prediction tasks.

As in the training phase, a particular advantage of the progress estimation is that all available data can be utilised without retraining the model. It is, for example, possible to estimate the DP if only cognitive scores are available for one visit and only image-based biomarkers for another visit. In this way, all available information can be employed in an intrinsic way.

### Outlook

While the presented method enables the combination of the information contained in all available measurements, it still relies on the definition of meaningful biomarkers. In this context, it would be interesting to employ biomarkers based on atrophy [49], tensor-based morphometry [13] or PET imaging [50], which could be integrated in a straightforward manner.

Currently, samples of subjects that are potentially suffering from terminal decline (36 of the 248 training subjects deceased during the study due to AD or another unknown cause) are not excluded from model training. While first experiments indicate a minor impact, it has not yet been analysed quantitatively if terminal decline impairs model training.

In the current approach for pre-alignment, only disease progress of the training subjects is aligned and not the progression rates. While this could be tackled in future versions of the model, preliminary experiments with an iterative pre-alignment techniques suggest that the effect is only marginal. Further, as discussed above, the pre-alignment introduces a potential bias in the progression models by considering the diagnosis and limiting the data pool to converters. In principle, however, the pre-alignment step is generic and any other suitable approach could be employed.

During progress estimation, the probabilities of all biomarkers values are assumed to be independent of each other, this means *f*_*Y*_(*y*_*sv*_1__, *y*_*sv*_2__|*τ*(***t***_*s*_)) = *f*_*Y*_(*y*_*sv*_1__|*τ*(*t*_*sv*_1__)) ⋅ *f*_*Y*_(*y*_*sv*_2__|*τ*(*t*_*sv*_2__)) for *v*_1_ ≠ *v*_2_. This is a simplification because the variation of biomarker values is hypothesised to be composed of inter-subject variability and noise. Accordingly, a value *y*_*sv*_2__ has a higher probability to lie on the quantile curve of *y*_*sv*_1__, which should be considered in a future model. Similarly, the independence across biomarkers is a simplified assumption, as dependencies between biomarkers exist, e.g. between ADAS 11 and ADAS13 scores.

In a related matter, intra-subject variability—which is particularly apparent for cognitive scores—is not explicitly modelled in the current approach. An enhanced modelling incorporating, for example, random effects would therefore be desirable.

A further challenge is extending the model domain *P* to a longer temporal span. In principle, the same approach could be employed to learn models for CN to MCI converters. A combination of the CN/MCI and MCI/AD models is therefore highly desirable and first steps in this direction have already been presented in [26]. Together with the use of further imaging biomarkers, this could potentially enhance the exploration of early disease progression, which is of particular clinical relevance because medication is most effective in the pre-symptomatic phase [13].

Besides prolonging the temporal span of the models, another approach would be to define a multi-dimensional progression domain *P*. This would relate, for example, to Stallard et al. [9], who split disease progression into cognitive, clinical, and functional progression. Similarly, incorporating age as a further covariate would allow a more realistic modelling compared to the currently implemented linear regression. However, these extensions would pose high demands on the size of the data set used for model training.

Given enough training data, it would also be highly interesting to generate specific models for certain subject groups, for example male and female or APoE-*ϵ*4 positive and negative subjects, which have a different age at disease onset [11]. Such personalised models could not only lead to a considerably improved progress estimation and allow further insights into the disease, but also address potential biases introduced by the temporal alignment.

Finally, the approach was only tested for analysing the progression of Alzheimer’s Disease. Potentially, other kinds of dementia or completely unrelated diseases could be regarded as well. The only requirement is the availability of meaningful biomarkers.

### Conclusion

In this work, an approach for biomarker-based disease progression modelling based on quantile regression was presented. The trained models were then employed to estimate the disease progress and the disease progression rate of test subjects. A main advantage of the method is that missing data is naturally handled, that means it can be employed even if the available biomarkers vary between the visits, without requiring a retraining of the model.

The presented experiments demonstrate the employment of the proposed method for disease estimation in several clinical applications, which range from classification over 4D atlas construction to the model-based prediction of biomarker values. The main focus of future work is on the extension of the model domain, which will allow a more precise estimation of early disease progression.

An open-source Python implementation of the presented method for progression modelling and the evaluation is available under https://github.com/aschmiri/DiseaseProgressionModel.

## Appendices

### A1 Model extrapolation

To extend the progression modelling to stages outside the interval *P* = [*p*^−^, *p*^+^], the model is extrapolated by extending the underlying predictor functions *η*(*p*) using exponential functions. These are chosen such that the derivatives at the end points *p*^±^ equal *η*′(*p*^±^). Further, scaling factors $\Delta_{\eta }^{\pm }$ defined by $\Delta_{\eta }^{\pm }:=\left|\eta \left(0\right)-\eta \left(p^{\pm }\right)\right|$ (i.e., depending on the amount of change of *η* in the MCI and AD intervals, respectively) are introduced, such that *η*(*p*) converges against $\eta \left(p^{\pm }\right)\pm \text{sgn}\left(\eta^{\prime }\left(p^{\pm }\right)\right)\Delta_{\eta }^{\pm }$ for *p* → ±∞. This results in sigmoid-like shaped predictor functions. The final formulation for $\tilde{\eta }\left(p\right)$ is then given by

$$\begin{array}{c} \tilde{\eta }\left(p\right):=\left\{ \begin{array}{cc} \eta \left(p^{-}\right)-\text{sgn}\left(\eta^{\prime }\left(p^{-}\right)\right)\Delta_{\eta }^{-}\left[1-\exp\left(\frac{|\eta^{\prime }\left(p^{-}\right)|}{\Delta_{\eta }^{-}}\left(p-p^{-}\right)\right)\right] & p<p^{-}\\ \eta (p) & p\in P\\ \eta \left(p^{+}\right)-\text{sgn}\left(\eta^{\prime }\left(p^{+}\right)\right)\Delta_{\eta }^{+}\left[\exp\left(\frac{|\eta^{\prime }\left(p^{+}\right)|}{\Delta_{\eta }^{+}}\left(p^{+}-p\right)\right)-1\right] & p>p^{+} \end{array}\right.. \end{array}$$

**Fig 12:**
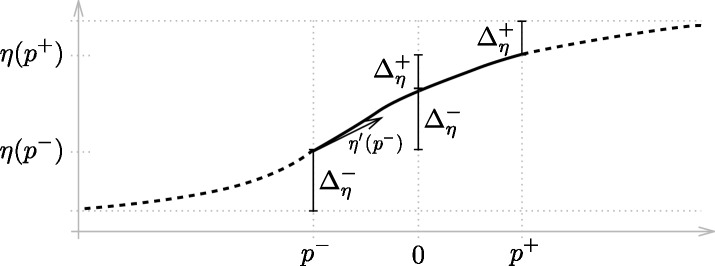
Illustration of the model extrapolation approach.

The model extrapolation is illustrated in A1 Figs 12 and 1C.

### A2 Brain structures

Volumes of the following 35 brain structures are considered as biomarkers *B*^vol^: 3rd Ventricle, 4th Ventricle, Left/Right Accumbens Area, Left/Right Amygdala, Brain Stem, Left/Right Caudate, Left/Right Cerebellum Exterior, Left/Right Cerebellum White Matter, Left/Right Cerebral White Matter, CSF, Left/Right Hippocampus, Left/Right Inferior Lateral Vent, Left/Right Lateral Ventricle, Left/Right Pallidum, Left/Right Putamen, Left/Right Thalamus Proper, Left/Right Ventral Diencephalon, Cerebellar Vermis, Left/Right Basal Forebrain, Left/Right Cortex.

## Supporting Information

S1 File ADNI Investigators. This file contains a complete list of ADNI investigators.
(PDF)
