# Supplementary material for: Learning Biomarker Models for Progression Estimation of Alzheimer’s Disease
Source: PLoS One. 2016 Apr 20;11(4):e0153040. doi: 10.1371/journal.pone.0153040 (PMC4838309; doi:10.1371/journal.pone.0153040)
Supplement: S1 File — This file contains a complete list of ADNI investigators. (PDF) [file pone.0153040.s001.pdf]

ADNI Alzheimer's Disease Neuroimaging Initiative

ACKNOWLEDGEMENT LIST FOR ADNI PUBLICATIONS

The Data and Publications Committee, in keeping with the publication policies adopted by the ADNI Steering Committee, here provide lists for standardized acknowledgement. The list consists of two parts: I. ADNI Infrastructure Investigators and Site Investigators and II. DOD ADNI Infrastructure Investigators and Site Investigators. Infrastructure Investigators represent the names responsible for leadership and infrastructure. Site Investigators represent the names of individuals at each recruiting site. All papers, including methodological papers, should have an acknowledgement list that consists of Infrastructure Investigators plus the FULL list.

I. ADNI I, GO and II

Part A: Leadership and Infrastructure

Principal Investigator

Michael W. Weiner, MD	UC San Francisco

ADCS PI and Director of Coordinating Center Clinical Core

Paul Aisen, MD	UC San Diego

Executive Committee

Michael Weiner, MD	UC San Francisco
Paul Aisen, MD	UC San Diego
Ronald Petersen, MD, PhD	Mayo Clinic, Rochester
Clifford R. Jack, Jr., MD	Mayo Clinic, Rochester
William Jagust, MD	UC Berkeley
John Q. Trojanowki, MD, PhD	U Pennsylvania
Arthur W. Toga, PhD	USC
Laurel Beckett, PhD	UC Davis
Robert C. Green, MD, MPH	Brigham and Women's Hospital/Harvard Medical School
Andrew J. Saykin, PsyD	Indiana University
John Morris, MD	Washington University St. Louis
Leslie M. Shaw	University of Pennsylvania

ADNI External Advisory Board (ESAB)

Zaven Khachaturian, PhD	Prevent Alzheimer's Disease 2020 (Chair)
Greg Sorensen, MD	Siemens
Maria Carrillo, PhD	Alzheimer's Association
Lew Kuller, MD	University of Pittsburg
Marc Raichle, MD	Washington University St. Louis
Steven Paul, MD	Cornell University
Peter Davies, MD	Albert Einstein College of Medicine of Yeshiva University
Howard Fillit, MD	AD Drug Discovery Foundation
Franz Hefti, PhD	Acumen Pharmaceuticals
Davie Holtzman, MD	Washington University St. Louis
M. Marcel Mesulam, MD	Northwestern University
William Potter, MD	National Institute of Mental Health
Peter Snyder, PhD	Brown University

ADNI 2 Private Partner Scientific Board (PPSB)

Adam Schwartz, MD	Eli Lilly (Chair)

Data and Publication Committee (DPC)

Robert C. Green, MD, MPH	BWH/HMS (Chair)

Resource Allocation Review Committee

Tom Montine, MD, PhD	University of Washington (Chair)

Clinical Core Leaders

Ronald Petersen, MD, PhD	Mayo Clinic, Rochester (Core PI)
Paul Aisen, MD	UC San Diego

Clinical Informatics and Operations

Ronald G. Thomas, PhD	UC San Diego
Michael Donohue, PhD	UC San Diego
Sarah Walter, MSc	UC San Diego
Devon Gessert	UC San Diego
Tamie Sather, MA	UC San Diego
Gus Jiminez, MBS	UC San Diego
Archana B. Balasubramanian, PhD	UC San Diego
Jennifer Mason, MPH	UC San Diego
Iris Sim	UC San Diego

Biostatistics Core Leaders and Key Personnel

Laurel Beckett, PhD	UC Davis (Core PI)
Danielle Harvey, PhD	UC Davis
Michael Donohue, PhD	UC San Diego

MRI Core Leaders and Key Personnel

Clifford R. Jack, Jr., MD	Mayo Clinic, Rochester (Core PI)
Matthew Bernstein, PhD	Mayo Clinic, Rochester
Nick Fox, MD	University of London
Paul Thompson, PhD	UCLA School of Medicine
Norbert Schuff, PhD	UCSF MRI
Charles DeCArli, MD	UC Davis
Bret Borowski, RT	Mayo Clinic
Jeff Gunter, PhD	Mayo Clinic
Matt Senjem, MS	Mayo Clinic
Prashanthi Vemuri, PhD	Mayo Clinic
David Jones, MD	Mayo Clinic
Kejal Kantarci	Mayo Clinic
Chad Ward	Mayo Clinic

PET Core Leaders and Key Personnel

William Jagust, MD	UC Berkeley (Core PI)
Robert A. Koeppe, PhD	University of Michigan

Norm Foster, MD	University of Utah
Eric M. Reiman, MD	Banner Alzheimer's Institute
Kewei Chen, PhD	Banner Alzheimer's Institute
Chet Mathis, MD	University of Pittsburgh
Susan Landau, PhD	UC Berkeley

Neuropathology Core Leaders

John C. Morris, MD	Washington University St. Louis
Nigel J. Cairns, PhD, MRCPath	Washington University St. Louis
Erin Householder	Washington University St. Louis
Lisa Taylor-Reinwald, BA, HTL (ASCP) – Past Investigator	Washington University St. Louis

Biomarkers Core Leaders and Key Personnel

Leslie M. Shaw, PhD	UPenn School of Medicine
John Q. Trojanowki, MD, PhD	UPenn School of Medicine
Virginia Lee, PhD, MBA	UPenn School of Medicine
Magdalena Korecka, PhD	UPenn School of Medicine
Michal Figurski, PhD	UPenn School of Medicine

Informatics Core Leaders and Key Personnel

Arthur W. Toga, PhD	USC (Core PI)
Karen Crawford	USC
Scott Neu, PhD	USC

Genetics Core Leaders and Key Personnel

Andrew J. Saykin, PsyD	Indiana University
Tatiana M. Foroud, PhD	Indiana University
Steven Potkin, MD UC	UC Irvine
Li Shen, PhD	Indiana University
Kelley Faber, MS, CCRC	Indiana University
Sungeun Kim, PhD	Indiana University
Kwangsik Nho, PhD	Indiana University

Initial Concept Planning & Development

Michael W. Weiner, MD	UC San Francisco
Lean Thal, MD	UC San Diego
Zaven Khachaturian, PhD	Prevent Alzheimer's Disease 2020

Early Project Proposal Development

Leon Thal, MD	UC San Diego
Neil Buckholtz	National Insititute on Aging
Michael W. Weiner, MD	UC San Francisco
Peter J. Snyder, PhD	Brown University
William Potter, MD	National Institute of Mental Health
Steven Paul, MD	Cornell Universtiy
Marylyn Albert, PhD	Johns Hopkins University
Richard Frank, MD, PhD	Richard Frank Consulting

Zaven Khachaturian, PhD — Prevent Alzheimer's Disease 2020

NIA
John Hsiao, MD — National Institute on Aging

Part B: Investigators By Site

Oregon Health and Science University:
Jeffrey Kaye, MD
Joseph Quinn, MD
Lisa Silbert, MD
Betty Lind, BS
Raina Carter, BA
Sara Dolen, BS – Past Investigator

University of Southern California:
Lon S. Schneider, MD
Sonia Pawluczyk, MD
Mauricio Beccera, BS
Liberty Teodoro, RN
Bryan M. Spann, DO, PhD – Past Investigator

University of California--San Diego:
James Brewer, MD, PhD
Helen Vanderswag, RN
Adam Fleisher, MD – Past Investigator

University of Michigan:
Judith L. Heidebrink, MD, MS
Joanne L. Lord, LPN, BA, CCRC

Mayo Clinic, Rochester:
Ronald Petersen, MD, PhD
Sara S. Mason, RN
Colleen S. Albers, RN
David Knopman, MD
Kris Johnson, RN – Past Investigator

Baylor College of Medicine:
Rachelle S. Doody, MD, PhD
Javier Villanueva-Meyer, MD
Munir Chowdhury, MBBS, MS
Susan Rountree, MD
Mimi Dang, MD

Columbia University Medical Center:
Yaakov Stern, PhD
Lawrence S. Honig, MD, PhD
Karen L. Bell, MD

Washington University, St. Louis:
Beau Ances, MD
John C. Morris, MD
Maria Carroll, RN, MSN
Mary L. Creech, RN, MSW
Erin Franklin, MS, CCRP
Mark A. Mintun, MD – Past Investigator
Stacy Schneider, APRN, BC, GNP – Past Investigator
Angela Oliver, RN, BSN, MSG – Past Investigator

University of Alabama - Birmingham:
Daniel Marson, JD, PhD
Randall Griffith, PhD, ABPP
David Clark, MD
David Geldmacher, MD
John Brockington, MD
Erik Roberson, MD
Marissa Natelson Love, MD

Mount Sinai School of Medicine:
Hillel Grossman, MD
Effie Mitsis, PhD

Rush University Medical Center:
Raj C. Shah, MD
Leyla deToledo-Morrell, PhD – Past Investigator

Wien Center:
Ranjan Duara, MD
Daniel Varon, MD
Maria T. Greig, MD
Peggy Roberts, CNA – Past Investigator

Johns Hopkins University:
Marilyn Albert, PhD
Chiadi Onyike, MD

Daniel D'Agostino II, BS
Stephanie Kielb, BS – Past Investigator

New York University:
James E. Galvin, MD, MPH
Brittany Cerbone
Christina A. Michel – Past Investigator
Dana M. Pogorelec – Past Investigator
Henry Rusinek, PhD – Past Investigator
Mony J de Leon, EdD – Past Investigator
Lidia Glodzik, MD, PhD – Past Investigator
Susan De Santi, PhD – Past Investigator

Duke University Medical Center:
P. Murali Doraiswamy, MBBS, FRCP
Jeffrey R. Petrella, MD
Salvador Borges-Neto, MD
Terence Z. Wong, MD – Past Investigator
Edward Coleman – Past Investigator

University of Pennsylvania:
Steven E. Arnold, MD
Jason H. Karlawish, MD
David Wolk, MD
Christopher M. Clark, MD

University of Kentucky:
Charles D. Smith, MD
Greg Jicha, MD
Peter Hardy, PhD
Partha Sinha, PhD
Elizabeth Oates, MD
Gary Conrad, MD

University of Pittsburgh:
Oscar L. Lopez, MD
MaryAnn Oakley, MA
Donna M. Simpson, CRNP, MPH

University of Rochester Medical Center:
Anton P. Porsteinsson, MD
Bonnie S. Goldstein, MS, NP
Kim Martin, RN
Kelly M. Makino, BS – Past Investigator
M. Saleem Ismail, MD – Past Investigator
Connie Brand, RN – Past Investigator

University of California, Irvine:
Ruth A. Mulnard, DNSc, RN, FAAN
Gaby Thai, MD
Catherine Mc-Adams-Ortiz, MSN, RN, A/GNP

University of Texas Southwestern Medical School:
Kyle Womack, MD
Dana Mathews, MD, PhD
Mary Quiceno, MD

Emory University:
Allan I. Levey, MD, PhD
James J. Lah, MD, PhD
Janet S. Cellar, DNP, PMHCNS-BC

University of Kansas, Medical Center:
Jeffrey M. Burns, MD
Russell H. Swerdlow, MD
William M. Brooks, PhD

University of California, Los Angeles:
Liana Apostolova, MD
Kathleen Tingus, PhD
Ellen Woo, PhD
Daniel H.S. Silverman, MD, PhD
Po H. Lu, PsyD – Past Investigator
George Bartzokis, MD – Past Investigator

Mayo Clinic, Jacksonville:
Neill R Graff-Radford, MBBCH, FRCP (London)
Francine Parfitt, MSH, CCRC
Tracy Kendall, BA, CCRP
Heather Johnson, MLS, CCRP – Past Investigator

Indiana University:
Martin R. Farlow, MD
Ann Marie Hake, MD
Brandy R. Matthews, MD
Jared R. Brosch, MD
Scott Herring, RN, CCRC – Past Investigator
Cynthia Hunt, BS, CCRP – Past Investigator

Yale University School of Medicine:
Christopher H. van Dyck, MD
Richard E. Carson, PhD
Martha G. MacAvoy, PhD
Pradeep Varma, MD

McGill Univ., Montreal-Jewish General Hospital:
Howard Chertkow, MD
Howard Bergman, MD
Chris Hosein, MEd

Sunnybrook Health Sciences, Ontario:
Sandra Black, MD, FRCPC
Bojana Stefanovic, PhD
Curtis Caldwell, PhD

U.B.C. Clinic for AD & Related Disorders:
Ging-Yuek Robin Hsiung, MD, MHSc, FRCPC
Howard Feldman, MD, FRCPC
Benita Mudge, BS
Michele Assaly, MA – Past Investigator

Cognitive Neurology - St. Joseph's, Ontario:
Elizabeth Finger, MD
Stephen Pasternack, MD, PhD
Irina Rachisky, MD
Dick Trost, PhD – Past Investigator
Andrew Kertesz, MD – Past Investigator

Cleveland Clinic Lou Ruvo Center for Brain Health:
Charles Bernick, MD, MPH
Donna Munic, PhD

Northwestern University:
Marek-Marsel Mesulam, MD
Kristine Lipowski, MASandra Weintraub, PhD
Borna Bonakdarpour, MD
Diana Kerwin, MD – Past Investigator
Chuang-Kuo Wu, MD, PhD – Past Investigator
Nancy Johnson, PhD – Past Investigator

Premiere Research Inst (Palm Beach Neurology):
Carl Sadowsky, MD
Teresa Villena, MD

Georgetown University Medical Center:
Raymond Scott Turner, MD, PhD
Kathleen Johnson, NP
Brigid Reynolds, NP

Brigham and Women's Hospital:
Reisa A. Sperling, MD
Keith A. Johnson, MD
Gad Marshall, MD

Stanford University:
Jerome Yesavage, MD
Joy L. Taylor, PhD
Barton Lane, MD
Allyson Rosen, PhD – Past Investigator
Jared Tinklenberg, MD – Past Investigator

Banner Sun Health Research Institute:
Marwan N. Sabbagh, MD
Christine M. Belden, PsyD
Sandra A. Jacobson, MD
Sherye A. Sirrel, CCRC

Boston University:
Neil Kowall, MD
Ronald Killiany, PhD
Andrew E. Budson, MD
Alexander Norbash, MD – Past Investigator
Patricia Lynn Johnson, BA – Past Investigator

Howard University:
Thomas O. Obisesan, MD, MPH
Saba Wolday, MSc
Joanne Allard, PhD

Case Western Reserve University:
Alan Lerner, MD
Paula Ogrocki, PhD
Curtis Tatsuoka, PhD
Parianne Fatica, BA, CCRC

University of California, Davis – Sacramento:
Evan Fletcher, PhD
Pauline Maillard, PhD
John Olichney, MD
Charles DeCarli, MD – Past Investigator
Owen Carmichael, PhD – Past Investigator

Neurological Care of CNY:
Smita Kittur, MD – Past Investigator

Parkwood Hospital:
Michael Borrie, MB ChB

T-Y Lee, PhD
Dr Rob Bartha, PhD

University of Wisconsin:
Sterling Johnson, PhD
Sanjay Asthana, MD
Cynthia M. Carlsson, MD, MS

University of California, Irvine - BIC:
Steven G. Potkin, MD
Adrian Preda, MD
Dana Nguyen, PhD

Banner Alzheimer's Institute:
Pierre Tariot, MD
Anna Burke, MD
Nadira Trncic, MD, PhD, CCRC
Adam Fleisher, MD – Past Investigator
Stephanie Reeder, BA – Past Investigator

Dent Neurologic Institute:
Vernice Bates, MD
Horacio Capote, MD
Michelle Rainka, PharmD, CCRP

Ohio State University:
Douglas W. Scharre, MD
Maria Kataki, MD, PhD
Anahita Adeli, MD

Albany Medical College:
Earl A. Zimmerman, MD
Dzintra Celmins, MD
Alice D. Brown, FNP

Hartford Hospital, Olin Neuropsychiatry Research Center:
Godfrey D. Pearlson, MD
Karen Blank, MD
Karen Anderson, RN

Dartmouth-Hitchcock Medical Center:
Laura A. Flashman, PhD
Marc Seltzer, MD
Mary L. Hynes, RN, MPH
Robert B. Santulli, MD – Past Investigator

Wake Forest University Health Sciences:
Kaycee M. Sink, MD, MAS
Leslie Gordineer
Jeff D. Williamson, MD, MHS – Past Investigator
Pradeep Garg, PhD – Past Investigator
Franklin Watkins, MD – Past Investigator

Rhode Island Hospital:
Brian R. Ott, MD
Henry Querfurth, MD
Geoffrey Tremont, PhD

Butler Hospital:
Stephen Salloway, MD, MS
Paul Malloy, PhD
Stephen Correia, PhD

UC San Francisco:
Howard J. Rosen, MD
Bruce L. Miller, MD
David Perry, MD

Medical University South Carolina:
Jacobo Mintzer, MD, MBA
Kenneth Spicer, MD, PhD
David Bachman, MD

St. Joseph's Health Care:
Elizabether Finger, MD
Stephen Pasternak, MD
Irina Rachinsky, MD
John Rogers, MD
Andrew Kertesz, MD – Past Investigator
Dick Drost, MD – Past Investigator

Nathan Kline Institute
Nunzio Pomara, MD
Raymundo Hernando, MD
Antero Sarrael, MD

University of Iowa College of Medicine
Susan K. Schultz, MD
Laura L. Boles Ponto, PhD
Hyungsub Shim, MD
Karen Ekstam Smith, RN

Cornell University
Norman Relkin, MD, PhD
Gloria Chaing, MD

Michael Lin, MD
Lisa Ravdin, PhD

University of South Florida: USF Health Byrd Alzheimer's Institute

Amanda Smith, MD
Balebail Ashok Raj, MD
Kristin Fargher, MD– Past Investigator

DOD ADNI

Part A: Leadership and Infrastructure

Principal Investigator

Michael W. Weiner, MD	University of California, San Francisco

ADCS PI and Director of Coordinating Center Clinical Core

Paul Aisen, MD	UC San Diego

Executive Committee

Michael Weiner, MD	UC San Francisco
Paul Aisen, MD	UC San Diego
Ronald Petersen, MD, PhD	Mayo Clinic, Rochester
Robert C. Green, MD, MPH	Brigham and Women's Hospital/ Harvard Medical School
Danielle Harvey, PhD	UC Davis
Clifford R. Jack, Jr., MD	Mayo Clinic, Rochester
William Jagust, MD	UC Berkeley
John C. Morris, MD	Washington University St. Louis
Andrew J. Saykin, PsyD	Indiana University
Leslie M. Shaw, PhD	Perelman School of Medicine, UPenn
Arthur W. Toga, PhD	USC
John Q. Trojanowski, MD, PhD	Perelman School of Medicine, University of Pennsylvania

Psychological Evaluation/PTSD Core

Thomas Neylan, MD	UC San Francisco

Traumatic Brain Injury/TBI Core

Jordan Grafman, PhD	Rehabilitation Institute of Chicago, Feinberg School of Medicine, Northwestern University

Data and Publication Committee (DPC)

Robert C. Green, MD, MPH	BWH/HMS (Chair)

Resource Allocation Review Committee

Tom Montine, MD, PhD	University of Washington (Chair)

Clinical Core Leaders

Michael Weiner MD	Core PI
Ronald Petersen, MD, PhD	Mayo Clinic, Rochester (Core PI)
Paul Aisen, MD	UC San Diego

Clinical Informatics and Operations

Ronald G. Thomas, PhD	UC San Diego
Michael Donohue, PhD	UC San Diego
Devon Gessert	UC San Diego
Tamie Sather, MA	UC San Diego
Melissa Davis	UC San Diego

Rosemary Morrison, MPH — UC San Diego
Gus Jiminez, MBS — UC San Diego

San Francisco Veterans Affairs Medical Center

Thomas Neylan, MD — UC San Francisco
Jacqueline Hayes — UC San Francisco
Shannon Finley — UC San Francisco

Biostatistics Core Leaders and Key Personnel

Danielle Harvey, PhD — UC Davis (Core PI)
Michael Donohue, PhD — UC San Diego

MRI Core Leaders and Key Personnel

Clifford R. Jack, Jr., MD — Mayo Clinic, Rochester (Core PI)
Matthew Bernstein, PhD — Mayo Clinic, Rochester
Bret Borowski, RT — Mayo Clinic
Jeff Gunter, PhD — Mayo Clinic
Matt Senjem, MS — Mayo Clinic
Kejal Kantarci — Mayo Clinic
Chad Ward — Mayo Clinic

PET Core Leaders and Key Personnel

William Jagust, MD — UC Berkeley (Core PI)
Robert A. Koeppe, PhD — University of Michigan
Norm Foster, MD — University of Utah
Eric M. Reiman, MD — Banner Alzheimer's Institute
Kewei Chen, PhD — Banner Alzheimer's Institute
Susan Landau, PhD — UC Berkeley

Neuropathology Core Leaders

John C. Morris, MD — Washington University St. Louis
Nigel J. Cairns, PhD, FRCPath — Washington University St. Louis
Erin Householder, MS — Washington University St. Louis

Biomarkers Core Leaders and Key Personnel

Leslie M. Shaw, PhD — Perelman School of Medicine, UPenn
John Q. Trojanowki, MD, PhD — Perelman School of Medicine, UPenn
Virginia Lee, PhD, MBA — Perelman School of Medicine, UPenn
Magdalena Korecka, PhD — Perelman School of Medicine, UPenn
Michal Figurski, PhD — Perelman School of Medicine, UPenn

Informatics Core Leaders and Key Personnel

Arthur W. Toga, PhD — USC (Core PI)
Karen Crawford — USC
Scott Neu, PhD — USC

Genetics Core Leaders and Key Personnel

Andrew J. Saykin, PsyD — Indiana University

Tatiana M. Foroud, PhD	Indiana University
Steven Potkin, MD UC	UC Irvine
Li Shen, PhD	Indiana University
Kelley Faber, MS, CCRC	Indiana University
Sungeun Kim, PhD	Indiana University
Kwangsik Nho, PhD	Indiana University

Initial Concept Planning & Development

Michael W. Weiner, MD	UC San Francisco
Karl Friedl	Department of Defense (retired)

Part B: Investigators By Site

University of Southern California:
Lon S. Schneider, MD, MS
Sonia Pawluczyk, MD
Mauricio Beccera

University of California, San Diego:
James Brewer, MD, PhD
Helen Vanderswag, RN

Columbia University Medical Center:
Yaakov Stern, PhD
Lawrence S. Honig, MD, PhD
Karen L. Bell, MD

Rush University Medical Center:
Debra Fleischman, Ph.D.
Konstantinos Arfanakis, Ph.D.
Raj C. Shah, M.D.

Wien Center:
Dr. Ranjan Duara MD PI
Dr. Daniel Varon MD Co-PI
Maria T Greig HP Coordinator

Duke University Medical Center:
P. Murali Doraiswamy, MBBS
Jeffrey R. Petrella, MD
Olga James, MD

University of Rochester Medical Center:
Anton P. Porsteinsson, MD (director)
Bonnie Goldstein, MS, NP (coordinator)
Kimberly S. Martin, RN

University of California, Irvine:
Ruth A. Mulnard, DNSc, RN, FAAN
Gaby Thai, MD
Catherine McAdams-Ortiz, MSN, RN, A/GNP

Medical University South Carolina:
Jacobo Mintzer, MD, MBA
Dino Massoglia, MD, PhD
Olga Brawman-Mintzer, MD

Premiere Research Inst (Palm Beach Neurology):
Carl Sadowsky, MD
Walter Martinez, MD
Teresa Villena, MD

University of California, San Francisco:
William Jagust MD
Susan Landau PhD
Howard Rosen, MD
David Perry

Georgetown University Medical Center:
Raymond Scott Turner, MD, PhD
Kelly Behan
Brigid Reynolds, NP

Brigham and Women's Hospital:
Reisa A. Sperling, MD
Keith A. Johnson, MD
Gad Marshall, MD

Banner Sun Health Research Institute:
Marwan N. Sabbagh, MD
Sandra A. Jacobson, MD
Sherye A. Sirrel, MS, CCRC

Howard University:
Thomas O. Obisesan, MD, MPH
Saba Wolday, MSc
Joanne Allard, PhD

University of Wisconsin:
Sterling C. Johnson, Ph.D.
J. Jay Fruehling, M.A.
Sandra Harding, M.S.

University of Washington:
Elaine R. Peskind, MD
Eric C. Petrie, MD, MS
Gail Li, MD, PhD

Stanford University:
Jerome A. Yesavage, MD
Joy L. Taylor, PhD
Ansgar J. Furst, PhD
Steven Chao, M.D.

Cornell University:
Norman Relkin, MD, PhD
Gloria Chaing, MD
Lisa Ravdin, PhD
